# CH2 Domain of Mouse IgG3 Governs Antibody Oligomerization, Increases Functional Affinity to Multivalent Antigens and Enhances Hemagglutination

**DOI:** 10.3389/fimmu.2018.01096

**Published:** 2018-05-23

**Authors:** Tomasz Klaus, Joanna Bereta

**Affiliations:** ^1^Laboratory of Monoclonal Antibodies, Małopolska Centre of Biotechnology, Jagiellonian University, Kraków, Poland; ^2^Department of Cell Biochemistry, Faculty of Biochemistry, Biophysics and Biotechnology, Jagiellonian University, Kraków, Poland

**Keywords:** IgG3, oligomerization, multivalent antigen, polyvalent antigen, hemagglutination, Ig constant region

## Abstract

Mouse IgG3 is highly protective against several life-threatening bacteria. This isotype is the only one among mouse IgGs that forms non-covalent oligomers, has increased functional affinity to polyvalent antigens, and efficiently agglutinates erythrocytes. IgG3 also triggers the complement cascade. The high efficacy of protection after passive immunization with IgG3 is correlated with the unique properties of this isotype. Although the features of IgG3 are well documented, their molecular basis remains elusive. Based on functional analyses of IgG1/IgG3 hybrid molecules with swapped constant domains, we identified IgG3-derived CH2 domain as a major determinant of antibody oligomerization and increased functional affinity to a multivalent antigen. The CH2 domain was also crucial for efficient hemagglutination triggered by IgG3 and was indispensable for complement cascade activation. This domain is glycosylated and atypically charged. A mutational analysis based on molecular models of CH2 domain charge distribution indicated that the functional affinity was influenced by the specific charge location. N-glycans were essential for CH2-dependent enhancement of hemagglutination and complement activation. Oligomerization was independent of CH2 charge and glycosylation. We also verified that known C1q-binding motifs are functional in mouse IgG3 but not in IgG1 framework. We generated for the first time a gain-of-function antibody with properties transferred from IgG3 into IgG1 by replacing the CH2 domain. Finding that the CH2 domain of IgG3 governs unique properties of this isotype is likely to open an avenue toward the generation of IgG3-inspired antibodies that will be protective against existing or emerging lethal pathogens.

## Introduction

There are four subclasses of mouse IgGs: IgG1, IgG2a, IgG2b, and IgG3. Although structurally very similar, they significantly differ in their functions (1). Mouse IgG3s are particularly interesting, because they are able to form oligomers, which strongly influences their biological activities (2).

Mouse IgG3 was described for the first time almost 50 years ago (3) and different aspects of its biology have been investigated by several groups. The propensity of IgG3 oligomerization was noticed already by its discoverers (3). Then, other researchers reported cooperative binding of IgG3 to a multivalent antigen (4, 5). Although the initial report on IgG3 oligomerization concerned molecules in solution (3), later studies revealed that binding to multivalent antigens promoted IgG3 intermolecular interactions, which in turn resulted in its increased functional affinity to the antigen (4). This phenomenon depended on Fc, because IgG3 F(ab’)_2_ fragments did not bind to the antigen cooperatively (4). However, the exact molecular mechanism of IgG3 oligomerization remains unknown.

IgG3 is a major component of cryoglobulins in mice (2). Cryoglobulins are plasma proteins that reversibly precipitate at low temperatures or at high concentrations (6). Cryogenic activity of IgG3 was shown to correlate with its ability to oligomerize, with the presence of charged residues in the variable region and the level of sialylation (7).

Also, IgG3 was reported as exceptionally effective in preventing or fighting several life-threatening microbial infections, e.g., with *Neisseria meningitidis* (8) or *Bacillus anthracis* (9). A comparison between the four mouse IgG subclasses with the same variable region specific to *B. anthracis* capsule proved that only IgG3 is protective against pulmonary anthrax in a mouse model (9). Importantly, mouse–human chimeric antibodies containing a constant region of any human IgG subclass were not effective, although they had the same variable region as the protective mouse IgG3 (10). These reports indicated that mouse IgG3 constant region has unique properties, but the authors only speculated about possible molecular mechanisms behind the observed phenomenon.

The exceptional characteristic of IgG3 was also confirmed by our previous report that among mouse IgGs recognizing a surface antigen of erythrocytes only IgG3s are able to agglutinate the cells (11). We rejected the hypothesis that IgG3-mediated hemagglutination results from oligomerization of the antibodies, because IgG3 F(ab’)_2_ was sufficient to trigger hemagglutination. Molecular modeling indicated that IgG3 has a larger span of Fab arms than other IgG subclasses. IgG3 has a long-upper hinge that may extend the Fab range, but whether this may account for the ability to hemagglutinate was not verified experimentally.

Here, we present the results of our attempts to find molecular determinants of the properties of mouse IgG3. Our experimental model comprised two antibodies (M18 and O10) specific to antigen B of the ABO blood group system (11). Antigen B is a pentasaccharide O-glycan attached to numerous proteins and lipids on the erythrocyte surface. The large quantity and high density of the antigen, as well as a strong negative charge of the erythrocyte surface, could be considered as a safe and easy-to-handle model of a pathogen surface. We generated many muteins of IgG3 molecules and analyzed their functional affinity to the antigen as well as their ability to hemagglutinate and oligomerize. The results showed that IgG3-derived CH2 domain determines antibody oligomerization and increases its affinity to the antigen. This domain also strongly enhances agglutination of erythrocytes bearing B antigen. Moreover, we investigated complement activation by the muteins, and we confirmed that known C1q-binding motifs are functional in mouse IgG3 but not in the mouse IgG1 framework.

## Materials and Methods

### Generation of Vectors Coding for Antibody Muteins

Expression vectors coding for heavy chain muteins with swapped domains or mutated CH2 were generated using synthetic nucleic acids (Gene Art, Germany) cloned into pFUSEss-CHIg-mG1_M18 (Addgene #82357) or pFUSEss-CHIg-mG3_M18 (Addgene #82356) plasmids (11). Only endogenous restriction sites present in the ORFs were used for cloning. Vectors coding for other muteins were prepared with Q5-based site directed mutagenesis kit (NEB). All plasmids coding for heavy chain variants of M18 antibody are available *via* Addgene repository along with their full sequences and maps, accession numbers: 105849–105863. Plasmids encoding O10 antibody variants were obtained by replacing the sequence coding for M18 variable fragment with a corresponding O10-derived cDNA using *EcoR*I and *Afe*I restriction sites in the vectors coding for M18 heavy chain variants. The sequence of O10 antibody is proprietary and cannot be disclosed. All plasmids were verified using Sanger sequencing (Genomed, Poland) or NGS (Addgene).

### Production of Antibodies

The recombinant antibodies were transiently expressed in HEK293T cells cultured in DMEM with 4.5 g/l glucose (Lonza) supplemented with 10% FBS (Biowest). The cells were co-transfected with plasmids coding for a heavy chain and a cognate light chain using Lipofectamine2000 (Thermo) or PEI MAX (Polysciences, MW 40,000). In the case of M18 variants, the plasmid pFUSE2ss-CLIg-mK_M18 (Addgene, #82358) (11) coding for M18 light chain was used. A similar plasmid coding for O10 light chain was used for expression of O10 variants. Hybridoma-derived M18 IgG3 was produced as described previously (11). The antibodies were purified using CaptureSelect LC-kappa (mur) affinity matrix (Thermo) according to instructions of the supplier. Glycine–HCl (100 mM, pH 2.0) was used for elution.

### Production of IgG3 F(ab’)_2_

A sequence encoding IgG2b core hinge, HA tag and a stop codon was cloned into pFUSEss-CHIg-mG3_M18 downstream of the sequence coding for the upper hinge of M18. Sequences of the upper and core hinges of the antibodies are described by Dangl et al (12). Mouse IgG2b core hinge contains four cysteine residues that allow an efficient association of Fab’ fragments into F(ab’)_2_ (13). Recombinant F(ab’)_2_ was expressed as described for other antibodies. Alternatively, F(ab’)_2_ was produced by enzymatic digestion with IdeZ (NEB) according to the manufacturer’s protocol.

### Measurements of Antibody Concentration

Antibody concentrations in cell culture media were measured using a standard sandwich ELISA on plates coated with sheep anti-mouse Fab polyclonal antibody (Jackson Laboratory, cat. #515-005-072, lot #105461). Goat anti-mouse kappa polyclonal antibody (1:3,000, BioRad, cat. #105008, batch #160617), HRP-labeled streptavidin (1:40,000, Sigma), and the TMB substrate for HRP (BD Bioscience) were used for detection. The HRP-dependent colorogenic reaction was stopped with 1 M HCl, and the absorbance at 450 nm was read using the microplate spectrophotometer Synergy H1 operated by Gen5 2.00 Software (BioTek). Purified M18 (IgG3) and MCP21 (IgG1, Sigma) were used as standards. Concentrations of muteins were calculated based on their Fab type (IgG1- or IgG3-type Fab). BCA assay (Sigma) was used for measurements of purified antibody concentrations with bovine γ-globulin (Thermo) as a reference.

### Antibody Binding to Immobilized Erythrocytes

Polystyrene plates were coated with 50 µg/ml poly-l-Lys (Sigma) for 1 h at room temperature (RT). Then, 100 µl of 0.1% (hematocrit) suspension of red blood cells in PBS were added to the wells and the cells were allowed to settle for 1 h at RT. After gentle aspiration of the solution, the cells were fixed using 0.025% glutaraldehyde for 40 min. Endogenous peroxidase activity was blocked with 3% H_2_O_2_ for 1 h. The plates were blocked overnight with 0.2% gelatin in PBS containing 0.05% Tween-20 at 4°C. Then, the cells were incubated with serial dilutions of cell culture media containing analyzed antibodies followed by detection with anti-mouse kappa polyclonal antibody conjugated with biotin (1:3,000, BioRad) and HRP-labeled streptavidin (1:40,000, Sigma). All reagents were diluted in the blocking buffer. The colorogenic reaction was performed, and the absorbance at 450 nm was measured as described above.

### C1q Binding

Duplicates of polystyrene plates were coated with 6 µg/ml of BSA conjugated with the discriminating trisaccharide of the B antigen (Dextra Laboratories, cat #NGP6323, batch #ATDX232-039) overnight at 4°C, blocked with 1% BSA in PBS (1 h) and incubated with serial dilutions of cell culture media containing O10 muteins (2 h). Then, one set of plates was used for evaluation of C1q binding by analyzed antibodies; and the plates were incubated with 2 µg/ml of C1q purified from human serum (Biorad, cat. #22215504, batch #130815; 2 h) and next with HRP-labeled anti-human C1q polyclonal antibody (1:400, Abcam, cat. #ab46191, lot #GR205436-5; 1 h). The second set of plates was used to analyze quantities of the muteins bound to the immobilized antigen; and the plates were incubated with anti-mouse kappa polyclonal antibody conjugated with biotin (1:3,000, BioRad, 2 h) and HRP-labeled streptavidin (1:40,000, Sigma; 1 h).

Absorbance of HRP product was measured as described above. Each incubation step was preceded by extensive washing with 0.05% Tween-20 in PBS. The antibodies, streptavidin, and C1q were diluted in 0.1% BSA in PBS. All incubations, except plate coating, were at RT.

The normalized C1q binding (*a*) was calculated by dividing the signal corresponding to C1q binding (*b*) by the signal corresponding to bound antibody (*c*).

$$\text{normalized }C1q\text{ binding}\left(a\right)=\frac{C1q\text{ binding signal}\left(b\right)}{\text{bound antibody signal}\left(c\right)}$$

Uncertainty of *a* (Δ*a*) was calculated by exact differential. Uncertainties of *b* and *c* (Δ*b* and Δ*c*) equaled to standard deviations of the absorbance measurements.

$$\text{uncertainty of }a\text{ ​ }\left(\Delta a\right)=\left|\frac{\partial a}{\partial b}\Delta b\right|+\left|\frac{\partial a}{\partial c}\Delta c\right|=\left|\frac{\Delta b}{c}\right|+\left|\frac{b}{c^{2}}\Delta c\right|$$

### Complement Cascade Activation

Washed human red blood cells suspended to a hematocrit of 2% were coated with 3 µg/ml or 1.5 µg/ml of the analyzed antibodies for 1.5 h at RT, washed twice with PBS and resuspended in PBS with Ca^2+^ and Mg^2+^. Then, the same volume of human complement serum (Sigma, cat. #S1764, lot #SLBS5471V, #SLBQ0752V or #SLBP0461V) diluted to 7 CH_50_ U/ml in PBS with Ca^2+^ and Mg^2+^ was added to the coated red blood cells. The samples were centrifuged after 2 h of incubation at 37°C, and the absorbance of released hemoglobin was measured in the supernatants at 540 nm.

### Red Blood Cells and Agglutination

Standard human red blood cells were purchased from Regional Centre of Blood Donation and Blood Treatment in Katowice, Poland. Agglutination tests were performed in 96-flat bottom plates. Serially diluted solutions of analyzed antibodies (100 µl) were gently mixed with 0.45% (hematocrit) suspension of red blood cells. The level of agglutination was analyzed using a phase-contrast microscope after 20 min of moderate shaking. A six-point scale was used for evaluation of agglutination intensity: from 4+ (complete cell aggregation), to 3+, 2+, 1+, ± to a negative score. The agglutination score reflects both the size of aggregates and quantity of non-agglutinated cells.

### IgG3 Self-Association Assay

Oligomerization of IgG3 was analyzed similarly to the method described by Abdelmoula et al (2). Three different concentrations of the purified domain muteins with M18 variable region (150, 100, and 20 µg/ml) were incubated with non-mutated, biotinylated IgG3 M18 (100 ng/ml) for 72–96 h at 4°C in the presence of 4% BSA (Sigma, cat. #A9576). Then, the antibody complexes were precipitated by adding 50% PEG-6000 (Sigma) to the final concentration of 7.5%. After 1 h of incubation on ice, the samples were centrifuged (30 min, 3,000 × *g*, 4°C). The supernatants were preserved for further analysis, and the precipitates were washed with ice-cold 7.5% PEG-6000 in PBS and centrifuged again (30 min, 3,000 × *g*, 4°C). Then, the precipitates were dissolved in PBS with 0.1% BSA by pipetting at 37°C. Biotinylated IgG3 in the precipitates and supernatants was quantified using ELISA on polystyrene plates coated with streptavidin (8 µg/ml, Thermo, cat. #434301, lot #RB233354). Bound biotinylated IgG3 was detected using rabbit monoclonal antibody M111-2 (1:1,000, Abcam, cat. #ab125904, lot #C050311, #GR157092-1) and HRP-labeled goat anti-rabbit polyclonal antibody (1:3,000, Sigma, cat #A6667). The absorbance of HRP product was measured as described above.

### SDS-PAGE and Western Blotting

Samples were resolved in 8 or 12% polyacrylamide gels under non-reducing or reducing conditions according to the protocol of Laemmli (14). After wet electrotransfer onto PVDF membrane and blocking with 4% skim milk in PBS, the samples were probed with anti-mouse kappa polyclonal antibody conjugated with biotin (1:3,000, BioRad) and HRP-labeled streptavidin (1:40,000, Sigma). Alternatively, rabbit anti-HA tag polyclonal antibody (1:10,000, Abcam, cat. #ab9110) and HRP-labeled goat anti-rabbit F(ab’)_2_ polyclonal antibody (1:10,000, Sigma, cat. #A6667, lot #SLBG3029) were used. Mouse IgG3 heavy chain was detected using goat antiserum to mouse IgG3 (1:500, Sigma, cat. #ISO2) and rabbit anti-goat polyclonal antibody (1:5,000, Sigma, cat. #A4174). Bands were visualized using Immobilon Western Chemiluminescent Substrate for HRP (Millipore). The images were captured and analyzed using Fusion Fx apparatus with the Fusion Capt Advance Fx5 program (Vilbert Lourmat, France).

## Results

### Comparison of Hemagglutination Induced by IgG3 and IgG3 F(ab’)_2_

**Figure 1:**
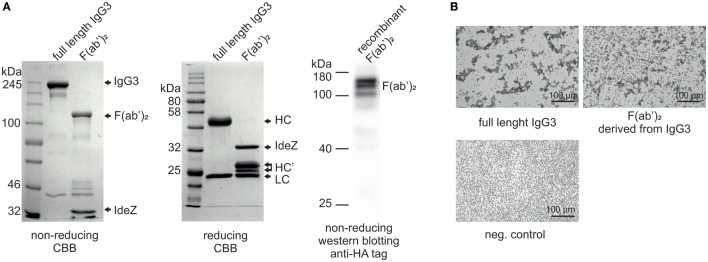
Hemagglutination induced by M18 IgG3 and its F(ab’)_2_. **(A)** Integrity of generated F(ab’)_2_ was verified using SDS-PAGE. In the case of the purified antibody digested with IdeZ, the gels were stained with Coomassie Brilliant Blue (CBB). Recombinant F(ab’)_2_ was equipped with HA tag and its integrity was confirmed using Western blotting with anti-HA tag antibody. The molecular mass of non-reduced F(ab’)_2_ is about 120 kDa. HC, heavy chain; HC’, heavy chain fragments generated after IdeZ cleavage; LC, light chain; **(B)** Microscopic images of erythrocytes agglutinated by equal molar concentrations of IgG3 antibody and its F(ab’)_2_. The antibody fragment was obtained from native IgG3 using IdeZ digestion.

In our previous work, we reported that F(ab’)_2_ obtained from IgG3 induces agglutination of erythrocytes bearing a cognate antigen (11). However, as shown below, complete IgG3 agglutinates erythrocytes more efficiently than its F(ab’)_2_, i.e., a much higher concentration of F(ab’)_2_ than that of the intact molecule is required for agglutination. We compared hemagglutination triggered by: (i) native, purified, full-length IgG3, and its F(ab’)_2_ obtained by IdeZ protease digestion and (ii) culture media of cells producing recombinant IgG3 or recombinant F(ab’)_2_ (Table 1). Concentrations of IgG3 and F(ab’)_2_ in the media were measured using ELISA and equalized for the comparative tests. We verified the quality of analyzed proteins and confirmed that recombinant IdeZ protease, which cleaves IgG3 at a single site in its hinge region, generates homogeneous F(ab’)_2_ (Figures 1A,B). Based on this analysis, we estimated that IgG3 was about 32 to 64-times more potent than its F(ab’)_2_ in hemagglutination (Table 1; Figure 1B). The results indicate that the Fc of IgG3 strongly enhances hemagglutination induced by this isotype.

**Table 1:** M18 full-length IgG3 and M18 IgG3 F(ab’)_2_ induced hemagglutination with different efficacy.

Concentration (nM)	Score of hemagglutination^a^		Concentration (nM)	Score of hemagglutination	
			
	Native IgG3	F(ab’)_2_ obtained using IdeZ		Recombinant IgG3	Recombinant F(ab’)_2_
82.5	++++	+	93	++++	±
41.3	++++	±	46.5	++++	–
20.6	++++	–	23.3	++++	–
10.3	++++	–	11.6	+++	–
5.2	+++	–	5.8	+++	–
2.6	+++	–	2.9	+	–
1.3	++	–	1.5	±	–
0.6	±	–	0.7	–	–
0.3	–	–	0.4	–	–

*^a^Representative results of three independent experiments*.

### The CH2 Domain Derived From IgG3 Enhanced Hemagglutination Efficacy of an Antibody

**Figure 2:**
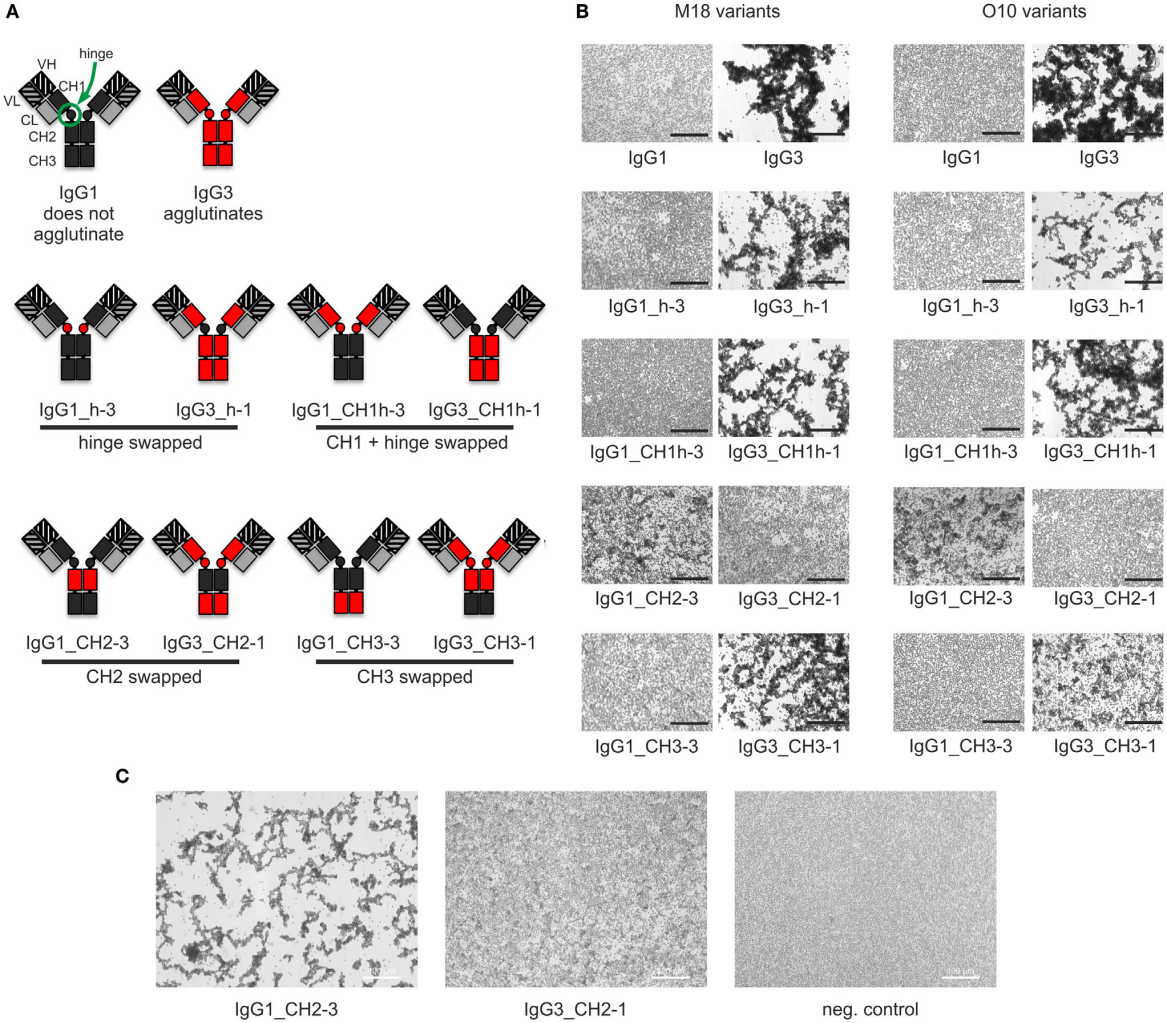
Hemagglutination induced by IgG1 and IgG3 muteins. **(A)** Generated domain muteins and their nomenclature. **(B)** Microscopic images of hemagglutination induced by the domain muteins. All antibodies were used at a concentration of 1.5 µg/ml, except of O10 IgG1_CH2-3 that was used at 3 µg/ml. Scale bar—100 µm; **(C)** Hemagglutination induced by selected O10 muteins used at 10 µg/ml.

Our previous attempts to explain the mechanism of IgG3-dependent hemagglutination brought us to the hypothesis that the elongated hinge of IgG3 determines its hemagglutination ability (11). In light of the new results, the hypothesis required revision. To elucidate which domains of IgG3 are crucial for its hemagglutination ability, we generated a panel of domain muteins of agglutinating IgG3 and non-agglutinating IgG1 isotypes (Figure 2A). We generated pairs of IgG1 and IgG3 molecules with the same variable regions and with swapped: (i) hinge regions; (ii) hinge regions + CH1 domains; (iii) CH2 domains, and (iv) CH3 domains and searched for muteins of two types: loss-of-function in the case of IgG3 and gain-of-function in the case of IgG1.

**Figure 3:**
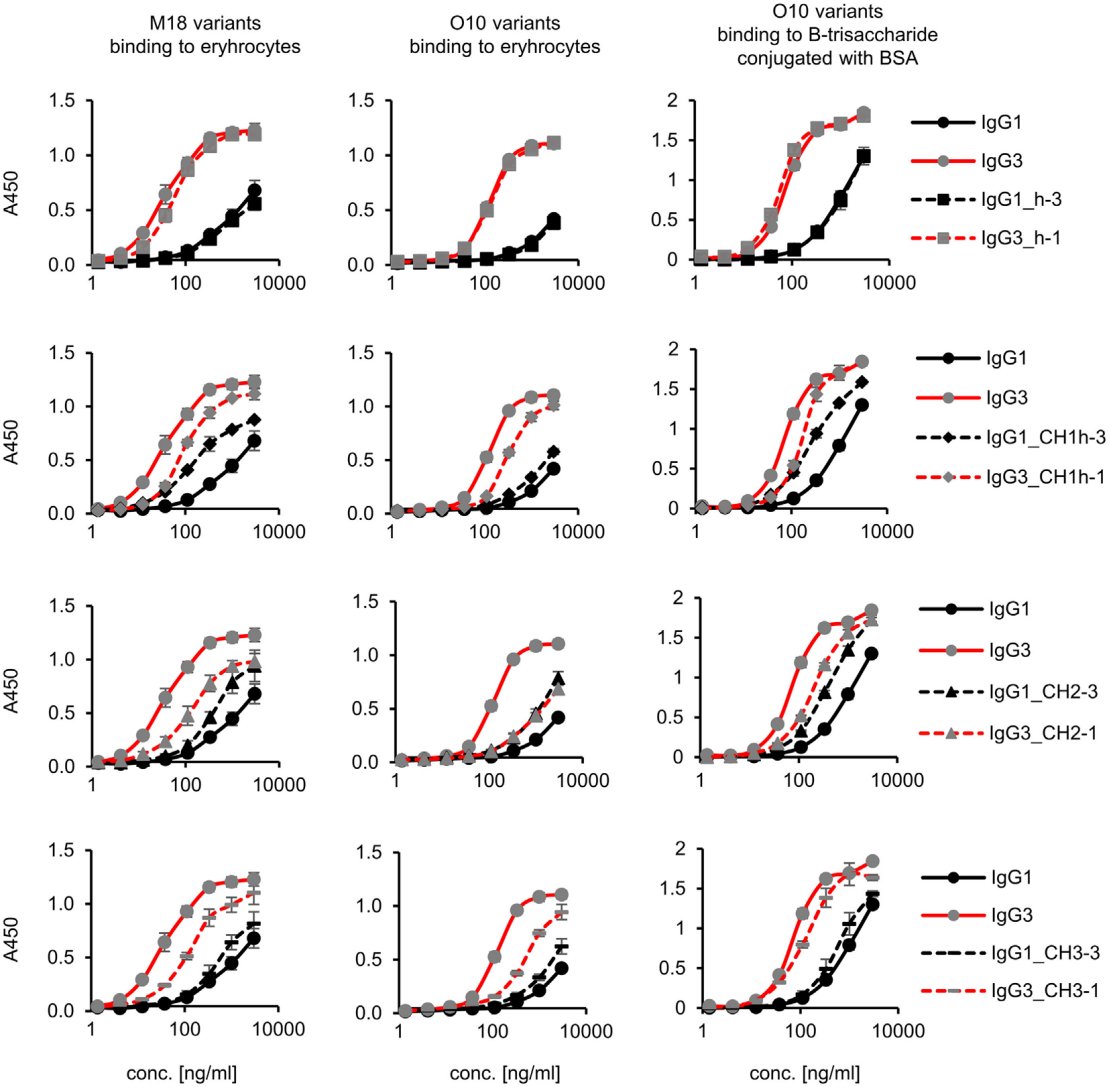
Antigen binding by the domain muteins. M18 and O10 antibodies are specific to B-antigen present on human erythrocytes. B-antigen is a pentasaccharide O-glycan. O10 antibody, but not M18, binds terminal fragment of the antigen, called B-trisaccharide. Antigen–antibody interaction was analyzed using ELISA on immobilized erythrocytes. In the case of O10, plates coated with BSA conjugated with the synthetic B-trisaccharide were also used. The plots present mean values from two independent experiments performed in duplicates. Results obtained for IgG1 and IgG3, the parental molecules, are presented on each plot to allow convenient comparisons.

Preliminary experiments showed that hinge swapping between IgG1 and IgG3 hinders disulfide bonds formation between chains of the muteins (Figure S1 in Supplementary Material). However, the IgG1_h-3 and IgG3_h-1 variants were functional and their affinity to the antigen was similar to that of the parental molecules (shown in Figure 3). As demonstrated by Dall’Acqua et al., immunoglobulins with modified hinges frequently form functional heterotetramers (HC)_2_(LC)_2_ despite the lack of disulfide bonds between the chains (15). Gel filtration confirmed that IgG1_h-3 and IgG3_h-1 have molecular mass greater than 150 kDa and form stable (HC)_2_(LC)_2_ heterotetramers (data not shown). To make sure that the results of the following experiments are not the consequence of incorrect assemblies of the hinge-swapped muteins, we also generated muteins with swapped fragments comprising hinge regions and CH1 domains. All muteins were successfully expressed and their integrity was verified using SDS-PAGE and Western blotting (Figure S1 in Supplementary Material).

Then, we compared hemagglutination induced by the muteins (Tables 2 and 3; Figures 2B,C). The results showed that neither the CH1 domain nor the hinge determined the agglutination ability of IgG3. The introduction of the CH3 domain from IgG1 into IgG3 resulted in a molecule with slightly reduced hemagglutination score but CH3 from IgG3 did not translate into IgG1 ability of hemagglutination. In contrast, CH2 swapping led to the generation of IgG1 mutein (IgG1_CH2-3) that gained the ability of hemagglutination (Tables 2 and 3). Moreover, the paired IgG3 mutein (IgG3_CH2-1) had about 16-times reduced hemagglutination score in comparison to the parental IgG3.

**Table 2:** Scores of hemagglutination induced by M18 variants.

Conc. (μg/ml)	Parental IgGs				Swap of hinge regions				Swap of CH1 + hinge domains				Swap of CH2 domains				Swap of CH3 domains			
					
	IgG1		IgG3		IgG1_h-3		IgG3_h-1		IgG1_CH1h-3		IgG3_CH1h-1		IgG1_CH2-3		IgG3_CH2-1		IgG1_CH3-3		IgG3_CH3-1	
									
	I^a^	II	I	II	I	II	I	II	I	II	I	II	I	II	I	II	I	II	I	II
1.500	–	–	+++	++	–	–	+++	+++	–	–	+++	+++	+	+	–	+	–	±	++	+++
0.750	–	–	+++	+++	–	–	+	+++	–	–	++	+++	–	±	–	–	–	–	+	++
0.375	–	–	++	+++	–	–	±	+	–	–	+	++	–	–	–	–	–	–	±	++
0.188	–	–	+	++	–	–	–	–	–	–	±	+	–	–	–	–	–	–	–	–
0.094	–	–	±	±	–	–	–	–	–	–	–	–	–	–	–	–	–	–	–	–
0.047	–	–	–	–	–	–	–	–	–	–	–	–	–	–	–	–	–	–	–	–

*^a^Results of two independent experiments designated as I and II*.

**Table 3:** Scores of hemagglutination induced by O10 variants.

Conc. (μg/ml)	Parental IgGs				Swap of hinge regions				Swap of hinge + CH1 domains				Swap of CH2 domains				Swap of CH3 domains			
					
	IgG1		IgG3		IgG1_h-3		IgG3_h-1		IgG1_CH1h-3		IgG3_CH1h-1		IgG1_CH2-3		IgG3_CH2-1		IgG1_CH3-3		IgG3_CH3-1	
										
	I^a^	II	I	II	I	II	I	II	I	II	I	II	I	II	I	II	I	II	I	II
3.000	–	–	++	++	–	–	+++	++	–	–	+++	++	+	+	–	±	–	+	+++	+++
1.500	–	–	+++	+++	–	–	+++	++	–	–	+++	++	±	–	–	–	–	–	+	++
0.750	–	–	++	++	–	–	++	++	–	–	+	++	–	–	–	–	–	–	±	±
0.375	–	–	+	+	–	–	+	+	–	–	±	±	–	–	–	–	–	–	–	–
0.188	–	–	±	±	–	–	±	±	–	–	–	+	–	–	–	–	–	–	–	–
0.094	–	–	–	–	–	–	–	–	–	–	–	–	–	–	–	–	–	–	–	–

*^a^Results of two independent experiments designated as I and II*.

IgG1_CH2-3 as a gain-of-function mutein was particularly interesting, because it indicated that the CH2 domain of IgG3 is the one critical for hemagglutination. However, IgG1_CH2-3 agglutinated erythrocytes with considerably lower score than native IgG3.

We also compared hemagglutination efficacy of native M18 IgG3 and its deglycosylated form. Deglycosylated IgG3 agglutinated erythrocytes about 16-times weaker than the native molecule (Table S1 in Supplementary Material).

To sum up, the ability of IgG3 to agglutinate erythrocytes results from its unique structure, in which the CH2 domain is especially important and strongly enhances the efficacy of the process. Although the IgG3 F(ab’)_2_ is sufficient to trigger hemagglutination, its efficacy is much lower in comparison to full-length IgG3, probably just due to the lack of the CH2 domain. The hinge region seems to have little influence on agglutination ability, because IgG3 with the IgG1-derived hinge agglutinated erythrocytes only slightly less effectively than the parental molecule.

### IgG3 Constant Domains Modify Functional Affinity to an Antigen

There is a general agreement that the increased functional affinity of IgG3 results from an avidity effect caused by the interactions between the Fc fragments of the molecules (16). In line with that, we observed that IgG3 binds to erythrocytes much more efficiently than IgG1 with the same variable region and much more efficiently than IgG3-derived F(ab’)_2_ (Figure S2 in Supplementary Material). Some authors discussed also the potential role of *N*-glycans in IgG3 unique properties (17, 18), but we did not observe any differences in antigen binding between control and deglycosylated antibody (Figure S2 in Supplementary Material).

Aiming to understand why IgG3 has increased functional affinity, we analyzed antigen binding by the domain muteins (Figure 3). The results showed that the hinge region of IgG3 does not influence the functional affinity of the antibody, but muteins with the swapped CH1 + hinge, CH2, or CH3 domains had changed functional affinity. The introduction of IgG3-derived CH1 + hinge or CH2 domain into the IgG1 framework enhanced antigen binding in comparison to the parental IgG1. Conversely, the paired IgG3 muteins with IgG1-derived CH1 + hinge or CH2 had reduced functional affinity. The swapping of the CH3 domains resulted in the IgG3 mutein with decreased affinity, but in the paired IgG1 mutein, the effect was not substantial. The calculated EC_50_ values of antigen binding for IgG3 muteins indicated that the CH2 domain had the strongest influence on IgG3-antigen interaction (Table 4). CH2 swapping resulted in IgG3 muteins with 3–12 times decreased functional affinity.

**Table 4:** EC_50_ of mutein binding to the antigen calculated using data from Figure 3.

Variable region of the mutein and type of the antigen	EC_50_ of mutein binding (nM)				
	
	IgG3	IgG3_h-1	IgG3_CH1h-1	IgG3_CH2-1	IgG3_CH3-1
M18 (erythrocytes)	0.24 ± 0.02	0.38 ± 0.01	0.61 ± 0.03	0.86 **±** 0.04	0.87 **±** 0.07
O10 (erythrocytes)	0.81 ± 0.01	0.88 ± 0.02	2.05 ± 0.06	9.75 **±** 2.04	3.60 ± 0.29
O10 (B-trisaccharide conjugated to BSA)	0.50 ± 0.04	0.38 ± 0.02	1.15 ± 0.06	1.40 **±** 0.03	0.79 ± 0.06

Overall, the results indicate that the higher (in comparison to IgG1) functional affinity of IgG3 to its antigen does not depend on a separate constant domain of this isotype, but rather is an additive result of discrete properties of the all three constant domains CH1, CH2, and CH3, but not the hinge region. Of all the constant domains, CH2 contributes the most to the high functional affinity of IgG3.

### Fc-Dependent Oligomerization of the Domain Muteins

The hallmark of mouse IgG3 is its ability to oligomerize. The process depends on Fc fragment, but its exact molecular mechanism is unknown. We analyzed whether the domain muteins form non-covalent complexes using polyethylene glycol (PEG) precipitation with a labeled IgG3 probe (2). In comparison to the original method, we used biotinylated IgG3 instead of radiolabeled IgG3. The IgG3-biotin interacted with oligomerizing muteins and the complexes comprised the mutein and the probe. The complexes were precipitated using PEG, and then, IgG3-biotin was quantified in precipitates and supernatants using ELISA. A high precipitate/supernatant ratio of IgG3-biotin quantities indicates that the mutein forms oligomers.

**Figure 4:**
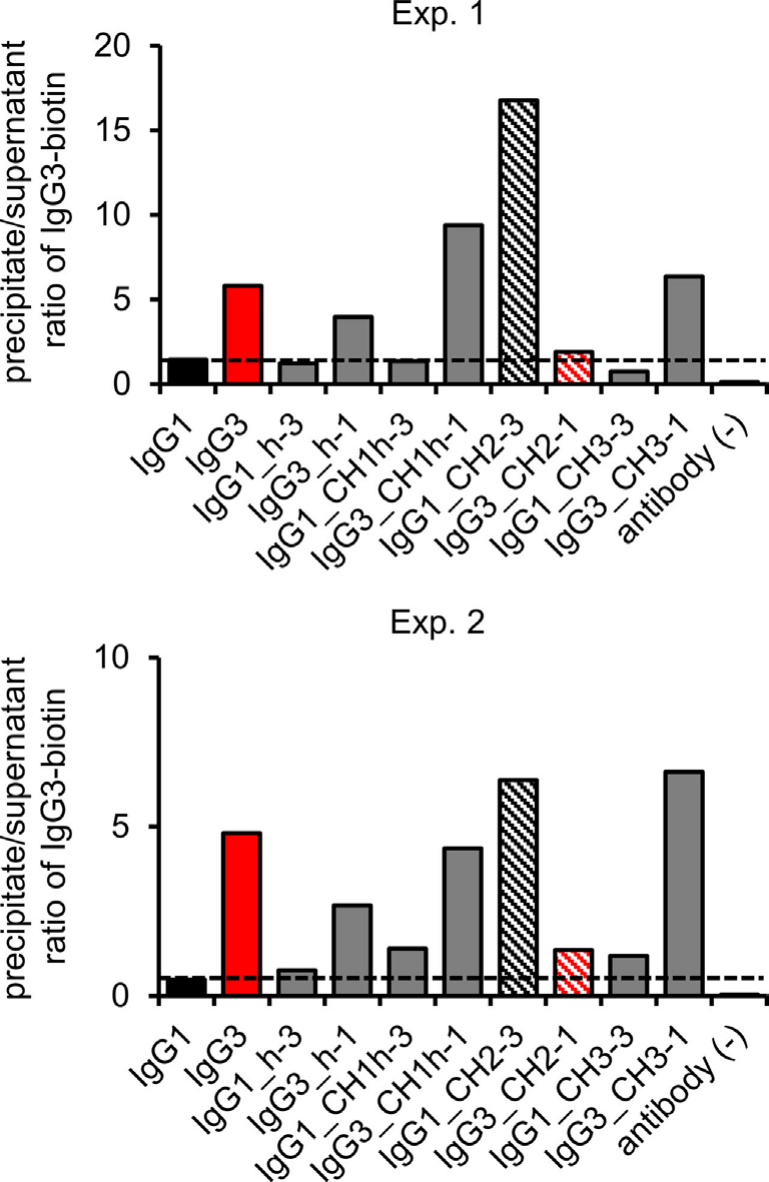
Oligomerization of the domain muteins. The antibodies (M18 variants, 150 µg/ml) were incubated at 4°C and oligomers were precipitated using PEG. Biotinylated IgG3 was used as a probe that oligomerized with the muteins and became a part of the complexes. The charts present results from two independent experiments. The results obtained for 100 and 20 µg/ml of the muteins are shown in Figure S3 in Supplementary Material. A percentage of the total IgG3-biotin detected in the precipitates and the supernatants is presented in Figure S4 in Supplementary Material.

The experiment showed that 5 out of 10 analyzed molecules form PEG-precipitable oligomers—IgG3 (control) and all IgG3 muteins but the one containing IgG1-derived CH2 domain and none of IgG1 muteins but the one with IgG3-derived CH2 domain (Figure 4). Oligomerization did not depend on CH2 glycosylation (Figure S5 in Supplementary Material). The results indicate that the CH2 domain is crucial for oligomerization of IgG3.

### Complement Activation by the IgG1 and IgG3 Muteins

Similar to human antibodies, there are pronounced differences between mouse IgG subclasses in their ability to trigger complement cascade. Mouse IgG3 activates complement efficiently, whereas mouse IgG1 does not. Although there are many excellent reports concerning correlation between human antibody structure and its ability of complement activation, the structural determinants of mouse antibodies that allow to trigger the cascade are not precisely known.

The best characterized Ig with respect to complement activation is human IgG1, in which several amino acid residues were identified as crucial for the initiation of the complement cascade (Figure S6 in Supplementary Material) (19–21). The sequence alignment of human IgG1, mouse IgG1, and mouse IgG3 indicated that the majority of human IgG1 amino acid residues involved in complement activation are conserved in both mouse isotypes (Figure S6 in Supplementary Material). However, it revealed two differences between mouse IgG1 and IgG3 within the regions corresponding to those involved in C1q binding by human IgG1—in the N-terminal fragment of the CH2 domain (Val231-Ser238 in IgG1 and Ile234-Pro238 in IgG3, EU numbering (22)) and in the residue 322 (Figure S6 in Supplementary Material).

**Figure 5:**
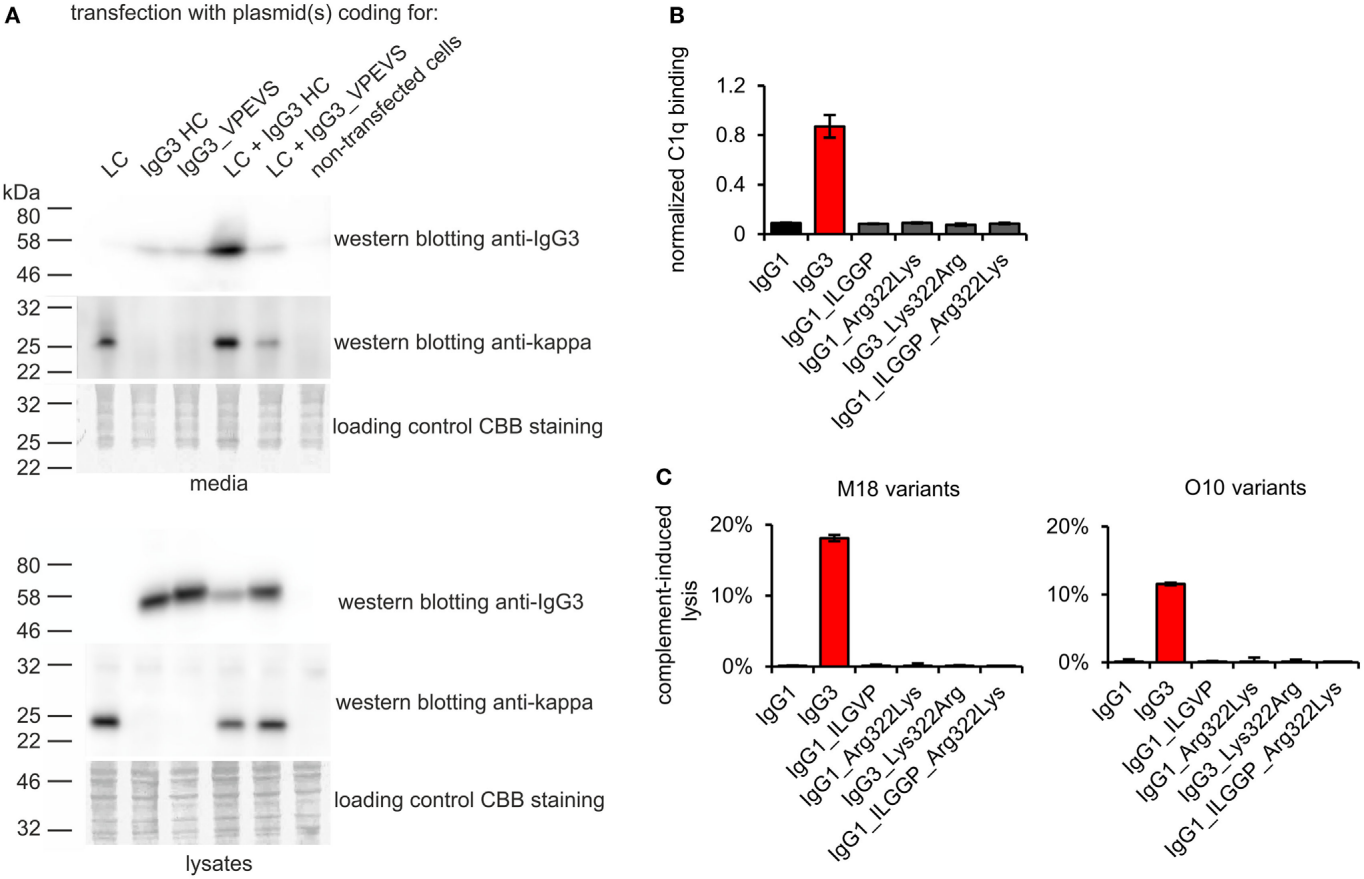
Functionality of known C1q-bining motifs in mouse IgG1 and mouse IgG3 frameworks. **(A)** IgG3_VPEVS did not associate with a light chain and was not secreted by the producing cells. **(B)** C1q binding by the muteins. Plates coated with BSA conjugated with the antigen, B-trisaccharide, were incubated with the muteins at 3 µg/ml (O10 variants). Then purified C1q was added. The muteins bind the antigen with different functional affinity. Thus, the C1q signal was normalized to the quantity of the bound antibody. Data used for calculation of the normalized binding are shown in Figure S7 in Supplementary Material. Error bars correspond to uncertainty calculated as presented in Section “Materials and Methods.” **(C)** Complement cascade activation by the muteins (3 µg/ml). Erythrocytes coated with the antibodies were incubated with complement serum. Complete lysis (100%) corresponds to water-induced lysis. In **(A–C)** representative results of two independent experiments are shown.

To verify whether these motifs are involved in complement activation by mouse IgG3, we generated additional muteins in which we swapped them between IgG1 and IgG3—IgG1_ILGGP (Val231Ile Pro232Leu Glu236Gly Val237Gly Ser238Pro); IgG3_VPEVS (Ile234Val Leu235Pro Gly236Glu Gly237Val Pro238Ser); IgG1_Arg322Lys; IgG3_Lys322Arg, and a double mutein IgG1_ILGGP_Arg322Lys. The IgG3 heavy chain containing VPEVS did not associate with a light chain and was not secreted (Figure 5A). Lys322Arg replacement completely abolished complement activation by IgG3 indicating that Lys322 is crucial for this process (Figures 5B,C). The three muteins of IgG1 did not bind C1q nor activated complement cascade indicating that the IgG1 framework prevents activation of complement (Figures 5B,C). The results showed that the known C1q-binding motifs are functional in the mouse IgG3 but not in the mouse IgG1 framework.

Some authors observed a correlation between hinge-dependent segmental flexibility of an antibody and its ability to activate complement (12). Thus, the differences between activity of mouse IgG1 and IgG3 are frequently explained on the basis of the length of their hinges. We decided to empirically verify this hypothesis using the domain muteins.

**Figure 6:**
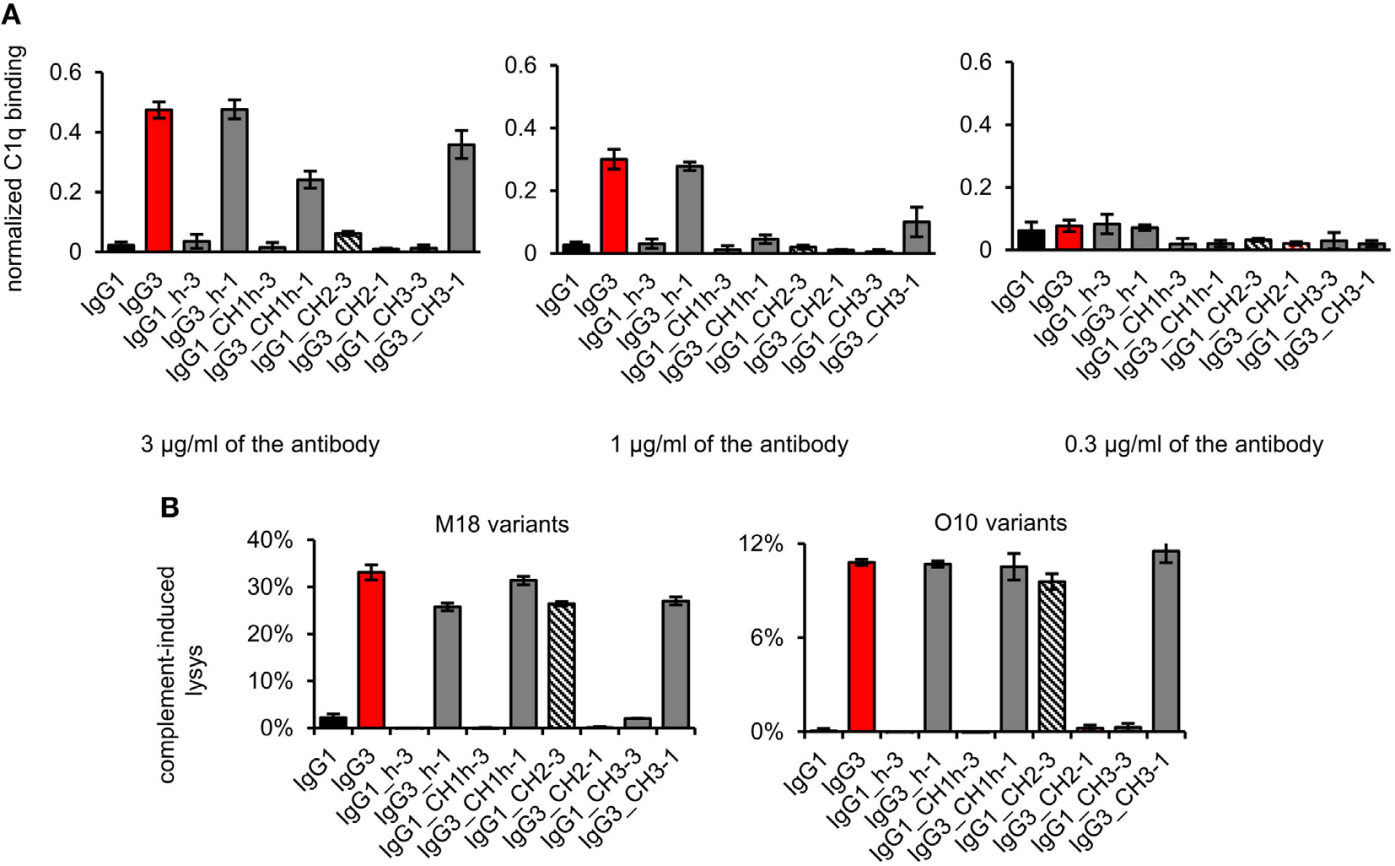
Complement activation induced by the domain muteins. **(A)** C1q binding to the domain muteins (O10 variants). The data used for calculations are presented in Figure S7 in Supplementary Material. Error bars correspond to uncertainty calculated as described in Section “Materials and Methods.” **(B)** Complement cascade activation by the domain muteins. Erythrocytes coated with 3 µg/ml of the muteins were incubated with complement serum. 100% lysis corresponds to water-induced lysis. The bars present mean values and standard deviation of duplicates from one experiment. Results obtained with 1.5 µg/ml of the muteins are presented in Figure S8 in Supplementary Material. **(A,B)** Representative results of two independent experiments.

First, we analyzed the binding of the complement cascade initiator (C1q) to the muteins (Figure 6A). The results showed that the hinge modification does not affect C1q binding. The swapping of the CH2 domain from IgG1 into IgG3 abolished C1q binding by the latter. Interestingly, the paired mutein (IgG1_CH2-3) did not gain the ability to strongly interact with C1q; its binding of C1q reached ~12% of that characteristic for native IgG3. Swapping of the CH1 + hinge domains or the CH3 domains between IgG1 and IgG3 moderately diminished C1q binding by IgG3 and did not increase its binding by IgG1.

We also analyzed complement activation in serum triggered by erythrocytes coated with the domain muteins (Figure 6B). The levels of erythrocyte lysis indicated that all muteins containing the CH2 domain derived from IgG3 activate complement cascade. The observed differences in C1q binding were not reflected by the different efficacy of the cascade triggering. The muteins with low (IgG1_CH2-3) or moderate (IgG3_CH1h-1, IgG3_CH3-1) ability of C1q-binding activated complement cascade with efficacy similar to that of the parental IgG3. It seems that in the case of the antibodies comprising IgG3-derived CH2 domain, even weak interaction with C1q was sufficient to effectively activate the whole complement cascade.

The results showed that both IgG1- and IgG3-derived CH1, hinge, and CH3 domains are permissive for C1q binding and complement activation. The CH2 domain of IgG1 is a non-permissive framework for the known C1q-binding motifs.

Overall, the results pointed to the CH2 domain as the major determinant of mouse IgG3 functions and unique properties of this isotype. In the last part of our work, we sought for features of the IgG3-derived CH2 domain that may account for IgG3 distinctive characteristic.

### Properties of Muteins With Reversed Charge of the CH2 Domains

The most striking difference between mouse IgG3-derived CH2 and CH2 domains of other IgG subclasses is their charges; only the former has a strong positive charge. For example, at pH 7.0, the net charge of the CH2 domain of IgG1 is −2.6 and of IgG3 is +2.6 (calculated using http://protcalc.sourceforge.net/). Hovenden et al. (9) found a correlation between the charge of CH2 domains of mouse IgG subclasses and their affinity to a negatively charged polyvalent antigen (poly-glutamic acid, poly-GA); and the high affinity of IgG3 to poly-GA was attributed to the charge of its CH2 domain.

**Figure 7:**
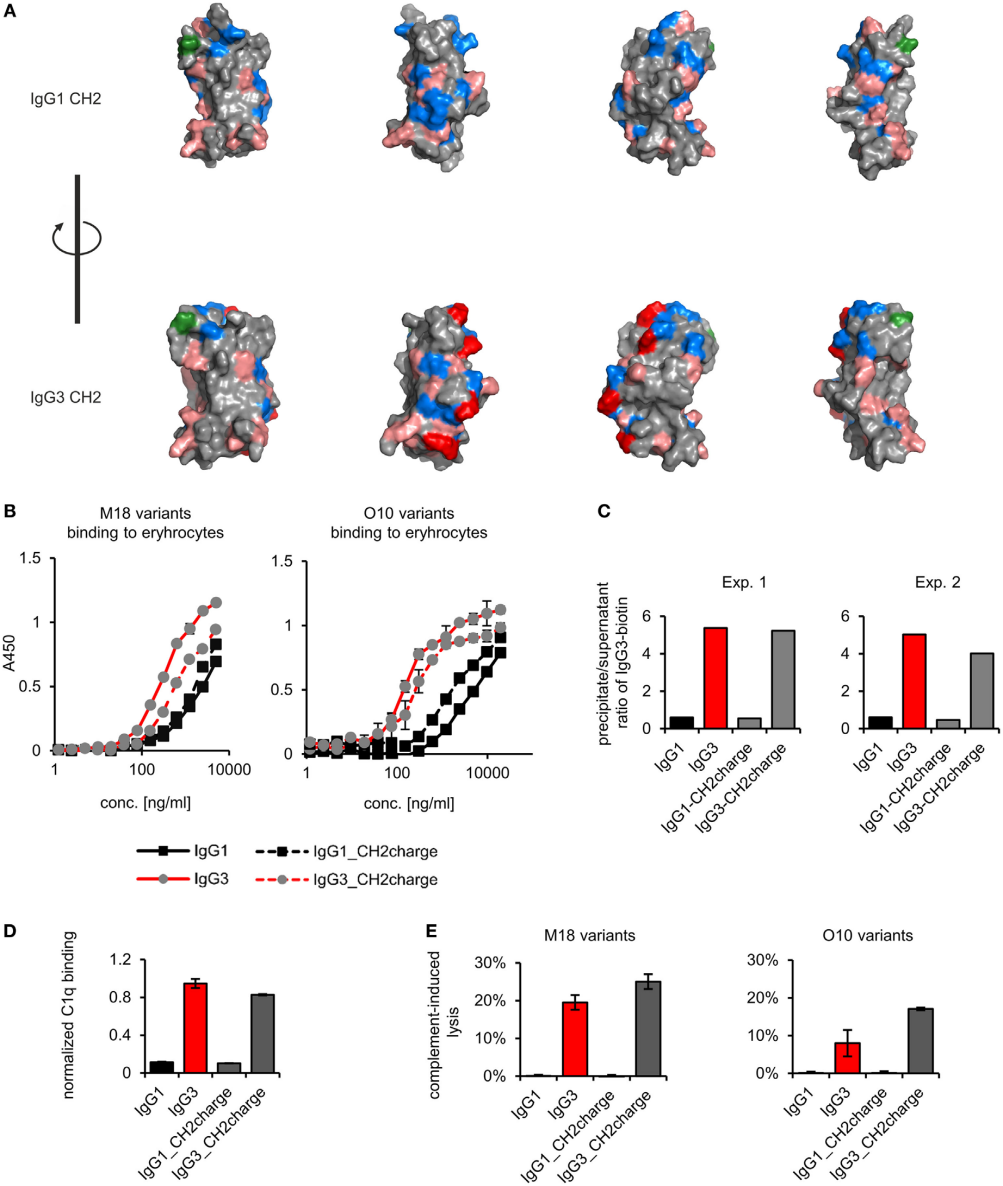
Properties of the muteins with modified charge of the CH2 domain. **(A)** Charge location on the CH2 domain of IgG1 and IgG3. Basic residues (Arg, His, and Lys) are faint red, acidic residues (Asp, Glu) are blue, and a site of CH2 N-glycosylation (Asn297) is green. His274, Lys282, Arg315, and Lys326 of IgG3 CH2 are dark red. These four residues were swapped between IgG1 and IgG3 to generate IgG1_CH2charge and IgG3_CH2charge muteins. The images present views obtained by 90° rotation of the domain models. **(B)** Antigen binding by the muteins. The charts present representative results of two independent experiments performed in duplicates or triplicates. Error bars equal to SD. **(C)** Oligomerization of the muteins. Results from two independent experiments with 100 µg/ml of the antibodies (M18 variants) are shown. A percentage of the total IgG3-biotin detected in precipitates and supernatants are presented in Figure S4 in Supplementary Material. Results for IgG1 and IgG3 are the same as in Figure 4 because the data were collected in the same experiments. **(D)** C1q binding by the muteins (O10 variants, 3 µg/ml). Data used for calculation of the normalized binding are shown in Figure S7 in Supplementary Material. The chart presents representative results of two independent experiments. Error bars correspond to uncertainty calculated as presented in Section “Materials and Methods.” **(E)** Complement cascade activation by the muteins (3 µg/ml). Erythrocytes coated with the antibodies were incubated with complement serum. Complete lysis (100%) corresponds to water-induced lysis. Representative results of two independent experiments are shown.

We analyzed spatial distribution of charged residues on the CH2 surface of IgG1 and IgG3 using previously obtained molecular models (11) and data deposited in PDB record 1IGY (Figure 7A). We identified 29 residues that differ between CH2 domains of mouse IgG1 and IgG3, 9 of which have different charge (Figure S9 in Supplementary Material). Based on the models, we selected four basic residues (His274, Lys282, Arg315, and Lys326) that are regularly spaced on the outer surface of the CH2 domain of IgG3 (Figure 7A; Figure S9 in Supplementary Material). The same residues in IgG1 are not charged. To verify whether CH2 charge influences IgG3 properties, we generated two muteins in which the four residues were swapped—IgG3_CH2charge (His274Gln Lys282Val Arg315Asn Lys326Ala) and IgG1_CH2charge (Gln274His Val282Lys Asn315Arg Ala326Lys). These muteins were expressed, correctly assembled, and soluble (Figure S1 in Supplementary Material). The introduced mutations reversed the charge of the CH2 domains. It was 0.6 and −0.7 at pH 7.0 for the CH2 domain of IgG1_CH2charge and IgG3_CH3charge, respectively.

We compared properties of the parental molecules and the muteins with modified CH2 charge. We observed that the charge influenced binding to erythrocytes (Figure 7B). However, hemagglutination, oligomerization, C1q binding, and complement activation were not affected by this charge modification (Table 5; Figures 7C–E). The results indicate that the four analyzed residues have only limited impact on the IgG3 properties. We cannot exclude that other charged residues within the CH2 domain of IgG3 may influence or determine properties of this isotype.

**Table 5:** Hemagglutination induced by the muteins with modified charge of the CH2 domain.

M18 variants					O10 variants				
	
Conc. (μg/ml)	IgG1	IgG3	IgG1_CH2charge	IgG3_CH2charge	Conc. (μg/ml)	IgG1	IgG3	IgG1_CH2charge	IgG3_CH2charge
5.00	±^a^	++++	–	+++	2.00	–	++++	±	++++
2.50	–	++++	–	+++	1.00	–	+++	–	+++
1.25	–	+++	–	++	0.50	–	++	–	++
0.63	–	++	–	++	0.25	–	±	–	±
0.31	–	+	–	±	0.13	–	–	–	–
0.16	–	±	–	–	0.06	–	–	–	–
0.00	–	–	–	–	0.00	–	–	–	–

*^a^Representative results of two independent experiments*.

## Discussion

We summarized the results of the experiments in Table 6. We observed that molecular determinants of the unique features of IgG3 are present in the CH2 domain. However, the modifications of CH2 differently affected the features suggesting that their molecular bases are different.

**Table 6:** Summary of experimental results.

IgG3 feature/function	Influence by the CH2 domain		
	
	Presence	Net charge^a^	Glycosylation
Hemagglutination	Strong enhancement	No effect	Enhancement
Functional affinity to polyvalent antigens	Strong enhancement	Weak to moderate effect	No effect
Oligomerization in solution	Dependence	No effect	No effect
Activation of complement cascade	Dependence	No effect	Dependence^b^

*^a^Associated with the presence of His274, Lys282, Arg315, Lys326*.

*^b^Data not shown*.

The prominent role of the CH2 domain in IgG3 biology was originally reported by Hovenden et al. (9). The authors investigated highly protective IgG3 antibodies against the capsular antigen of *B. anthracis*. They generated an IgG3 mutein with CH2 swapped from non-protective IgG2b. The mutein lost protective activity of the parental molecule and had reduced affinity to the antigen. In contrast to the work of Hovenden et al., we generated, for the first time, an antibody mutein that gained the unique properties of IgG3. We swapped IgG3-derived CH2 into IgG1, and the obtained molecule (IgG1_CH2-3) had properties typical for IgG3—it agglutinated erythrocytes, oligomerized, had increased functional affinity to a polyvalent antigen, and activated the complement cascade. Thus, we proved that these unique features of mouse IgG3 could be transferred into a new antibody framework.

The mechanism of IgG3-dependent hemagglutination is still not completely understood. We previously reported that F(ab’)_2_ of IgG3 is sufficient to agglutinate erythrocytes (11). Here, we show that the presence of the CH2 domain in the IgG3 molecule profoundly diminishes the antibody concentration required for the F(ab’)_2_-mediated process. Moreover, the introduction of IgG3-derived CH2 into IgG1 framework resulted in the IgG1_CH2-3 mutein that agglutinates erythrocytes. The results indicate that efficient hemagglutination is triggered only by the antibodies equipped with the IgG3-derived CH2 domain.

The CH2 domain of IgG3 is positively charged at neutral pH. In contrast, the CH2 domains of other IgG subclasses are negatively charged under the same condition. Considering that erythrocyte surface has a strong negative charge and high zeta potential, it was likely that a positive charge of the IgG3-derived CH2 domain reduces the zeta potential and as a consequence enhances hemagglutination. Unexpectedly, net charge modification of the CH2 domains in IgG1 and IgG3 did not change hemagglutination potential of these isotypes, and we had to reject the hypothesis linking the CH2 net charge with the efficiency of hemagglutination.

Alternatively, antibody oligomerization may explain hemagglutination enhancement by the CH2 domain of IgG3. We showed that this domain solely determined antibody oligomerization in solution and thus most probably also on a multi-epitope surface. It is possible that oligomerization between antibodies bound to separate erythrocytes occurs parallel to a sensitization phase of hemagglutination. Thus, antibody oligomerization may lead to the formation of zipper-like structures that stabilize cell aggregates and increase a hemagglutination score. Moreover, the CH2 domain of IgG3 increased functional affinity of an antibody to erythrocyte surface. Thus, hemagglutination enhancement may at least partially depend on the increased affinity.

However, the observed enhancement of hemagglutination by the CH2 domain of IgG3 was affected by enzymatic deglycosylation. In contrast, oligomerization in solution and increased functional affinity to polyvalent antigen were independent of CH2 glycosylation. This difference indicates that antibody oligomerization does not fully account for the CH2 domain-mediated enhancement of hemagglutination.

Mouse IgG3 has a putative site of N-glycosylation in its CH3 domain on Asn471. Panka reported that the mutation of this Asn residue into Ser diminished the self-association of IgG3 (17). This finding was later contradicted by Kuroki et al., who provided evidence that this putative N-glycosylation site in the CH3 domain is not occupied and the mutation Asn471Thr does not influence IgG3 self-association or cryoglobulin activity (18). Our observations are in line with the findings of Kuroki et al. We did not observe any differences between oligomerization of IgG3 and its enzymatically deglycosylated variant. It is important to note that we and Kuroki et al. used PEG-precipitation for oligomerization analyses. Panka used different methods, ELISA and native electrophoresis, which may account for the discrepancies.

Greenspan et al. showed that Fc-dependent oligomerization increases functional affinity of IgG3 to polyvalent antigens (5). Our results confirm that finding, but we showed that the relation between oligomerization and increased functional affinity is more complex than previously thought. First, functional affinity of IgG3 was influenced not only by Fc region (CH2 and CH3 domains) but also by the CH1 domain. Second, functional affinity to the polyvalent antigen (B antigen) was modulated by the CH2 charge. In contrast, oligomerization in solution required only the presence of the CH2 domain of IgG3 and was insensitive to the introduced charge modifications. The results showed that the mechanism behind high functional affinity may depend on more factors than oligomerization in solution does.

The observed influence of the CH1 domain on functional affinity is difficult to explain. The CH1 domain of IgG3 has a more positive net charge than the CH1 domain of IgG1 (9). It is likely that the net charge of the CH1 domain influences the binding of the domain muteins to erythrocytes, which have a strong negative charge. However, the IgG3-derived CH1 domain also enhanced the binding of IgG1_CH1-3 to a surface with the immobilized trisaccharide B-BSA conjugate. Thus, the results support previous observations (23) that the CH1 domain may influence a variable domain and a paratope of an antibody.

According to the general view, the Fab and Fc fragments are independent parts of an antibody (24). However, our results demonstrate that the Fc, particularly its CH2 domain, may influence Fab-mediated antigen binding. There are two possible mechanisms of this phenomenon—intramolecular signaling (25) [called by some authors as an intramolecular allostery (16)] or intermolecular cooperativity.

There are several examples of intramolecular signaling observed by different authors investigating how the isotype switching changes an antibody affinity to its antigen [reviewed in Ref. (16, 26)]. The effects of the CH1 domains or Fc fragments on variable regions are well documented, but considered a rather unique phenomenon (16). It is more likely that the increased affinity of IgG3 to its antigen results from cooperativity of its CH2 domains. Within this domain, a specific site of self-association may be present, which governs oligomerization of an antibody and pre-determines the increased affinity to multivalent antigens. However, we cannot exclude other scenarios—the involvement of both the CH2 and CH3 domains in IgG3 intermolecular interactions or even sole CH3-CH3 interactions, assuming that the CH2 domains influence the whole molecule structure and promote reciprocal interactions of the CH3 domains of neighboring molecules.

Other factors, e.g., influence of the CH1 domain on a paratope, properties of an antigen (charge), spatial distribution of epitopes, intermolecular forces between epitope and paratope, or a variable domain framework may further modulate functional affinity of IgG3 upon multivalent antigen binding.

Diebolder at al. described recently an interesting example of Fc-dependent antibody oligomerization. Analyses of antibody binding to DNP-labeled liposomes (a multivalent antigen) revealed that human IgG may form hexamers through non-covalent interactions between their constant regions (27). Several mutations that enhance these interactions and subsequent complement activation were reported (27). The Fc-interactions promoting antibody hexamerization did not change affinity to the cognate antigen. Thus, this phenomenon seems to be different from IgG3 oligomerization, and it is still an open question whether mouse antibodies are able to form such hexamers.

Currently, no structure is available for a full-length mouse IgG3 or its Fc fragment. We performed some analyses using a molecular model of IgG3 obtained by comparative modeling, but its resolution is not sufficient for in-depth studies. IgG3 crystallization might provide a direct insight into the mechanism of its oligomerization, as was in the case of human IgG1 hexamerization described in the cited work (27).

Complement cascade activation, as an effector function of antibodies, constitutes a first-line of defense against microbial infections. As the cascade progresses, components of the complement are deposited on a pathogen surface and act as opsonins for phagocytic cells. Moreover, the complement lyses invading pathogens by forming membrane attacking complex. We confirmed that C1q-binding motifs, known from human IgG1, are functional in the mouse IgG3 framework. On the other hand, we did not observe complement activation by mouse IgG1 equipped with the motifs. The results indicate that the presence of the known C1q-binding motifs is not sufficient for complement activation by an antibody. The motifs must be surrounded by a permissive framework, provided e.g., by human IgG1 or mouse IgG3.

Our work suggests that a novel type of monoclonal antibodies may be generated by replacing the CH2 domain of a human antibody with the homologs fragment of mouse IgG3. Human IgG1 subclass is the most feasible target framework for generation of such IgG3-inspired hybrid mouse/human molecule (28). Our observation indicates that the generated hybrid antibody should preserve the ability to activate complement and may have increased affinity to polyvalent antigens. Since the mouse IgG3 subclass is highly protective against several life-threatening microbial infections, the hybrid molecule may be very useful in preventing or fighting lethal pathogens. However, the hybrid antibody with the mouse CH2 may be immunogenic. To decrease the risk of an unwanted immune response, the mouse component should be reduced to a minimum. Thus, the properties of the CH2 domain derived from mouse IgG3 should be further investigated and efforts should be made especially to identify fragments of this domain that determines its properties.

## Author Contributions

TK conceived and did all experiments. TK and JB analyzed and discussed the results. The manuscript was written by TK and JB. The authors accepted the final version of the manuscript.

## Conflict of Interest Statement

The authors declare that the research was conducted in the absence of any commercial or financial relationships that could be construed as a potential conflict of interest.
