# Supplementary material for: CH2 Domain of Mouse IgG3 Governs Antibody Oligomerization, Increases Functional Affinity to Multivalent Antigens and Enhances Hemagglutination
Source: Front Immunol. 2018 May 23;9:1096. doi: 10.3389/fimmu.2018.01096 (PMC5974032; doi:10.3389/fimmu.2018.01096)
Supplement: Supplementary file 1 [file data_sheet_1.DOCX]

CH2 domain of mouse IgG3 governs antibody oligomerization, increases functional affinity to multivalent antigens and enhances hemagglutination

Tomasz Klaus^1,2^, Joanna Bereta^2*^

^1^Laboratory of Monoclonal Antibodies, Małopolska Centre of Biotechnology, Jagiellonian University in Kraków, Poland

^2^Department of Cell Biochemistry, Faculty of Biochemistry, Biophysics and Biotechnology, Jagiellonian University in Kraków, Poland

*** Correspondence:**Joanna Bereta
address: Department of Cell Biochemistry, Faculty of Biochemistry, Biophysics and Biotechnology, Jagiellonian University in Kraków, Gronostajowa 7, 30-387 Kraków, Poland
e-mail: [joanna.bereta@uj.edu.pl](mailto:joanna.bereta@uj.edu.pl)
phone: +48-12-664-6356

Supplementary Figures and Tables


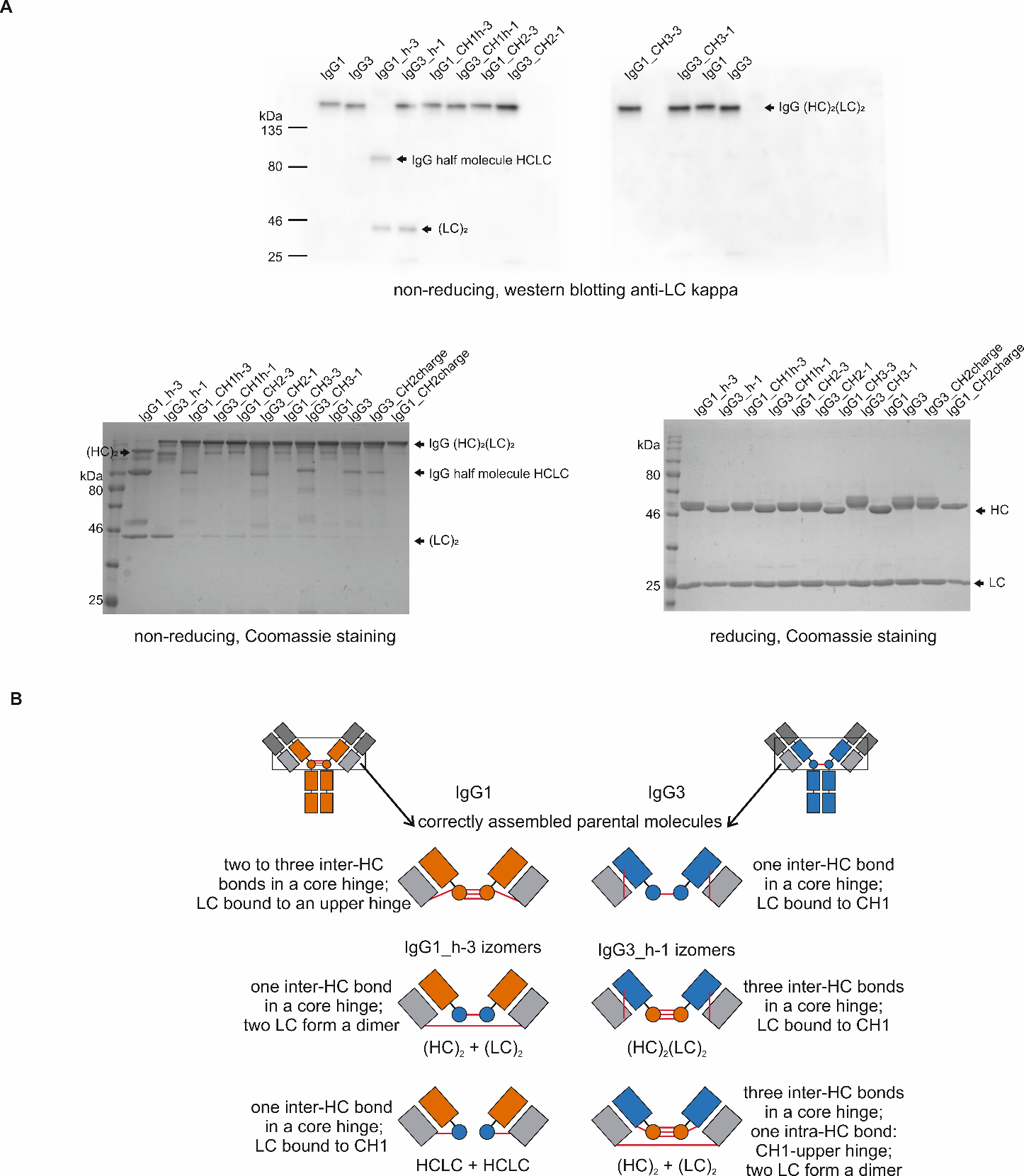


**Supplementary Figure 1** – Integrity of the domain muteins. (**A**) Samples of media collected from cell cultures producing the muteins were analyzed using western blotting with anti-kappa LC antibody. Purified antibodies were resolved using SDS-PAGE under non-reducing or reducing conditions. The gels were stained with Coomassie Brilliant Blue. (**B**) Influence of the hinge region on the molecule assembly.

**Supplementary Table 1** – Comparison of hemagglutination induced by native and deglycosylated IgG3.

|  | Experiment 1 | | Experiment 2 | |
| --- | --- | --- | --- | --- |
| Conc. [μg/ml] | Native M18 IgG3 | Deglycosylated M18 IgG3* | Native M18 IgG3 | Deglycosylated M18 IgG3 |
| 333.3 | ++++ | +++ | +++ | ++ |
| 166.7 | ++++ | +++ | +++ | ++ |
| 83.3 | ++++ | ++ | +++ | ++ |
| 41.7 | ++++ | ++ | +++ | ++ |
| 20.8 | ++++ | + | +++ | ++ |
| 10.4 | ++++ | + | +++ | ++ |
| 5.2 | +++ | +/- | +++ | + |
| 2.6 | ++ | - | ++ | +/- |
| 1.3 | ++ | - | ++ | - |
| 0.7 | + | - | + | - |
| 0.3 | - | - | - | - |
| 0 | - | - | - | - |

*M18 was deglycosylated using PNGase F (Promega) according to the manufacturer’s protocol. Deglycosylation was confirmed by a band shift to a lower molecular mass in SDS-PAGE (data not shown).


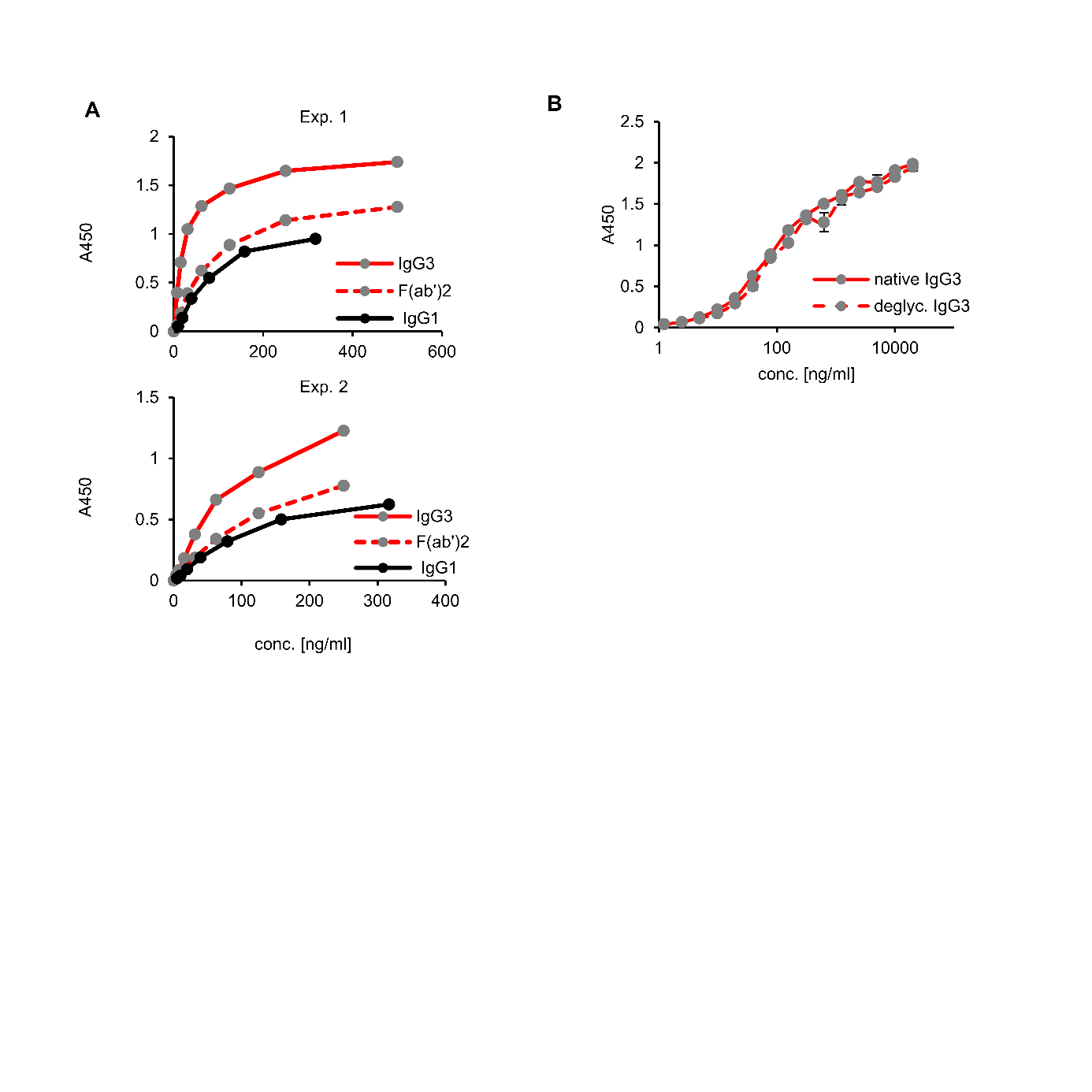


**Supplementary Figure 2** – Antigen-antibody interaction analyzed using ELISA on immobilized erythrocytes. (**A**) Antigen binding by IgG3, IgG3 F(ab’)_2_, and IgG1 switch variants. All antibodies had M18 variable fragment. F(ab’)_2_ was obtained by pepsin digestion. (**B**) Antigen binding by native and deglycosylated M18. Deglycosylation was performed using PNGase F (Promega) and confirmed by a band shift in SDS-PAGE analysis (data not shown).

**
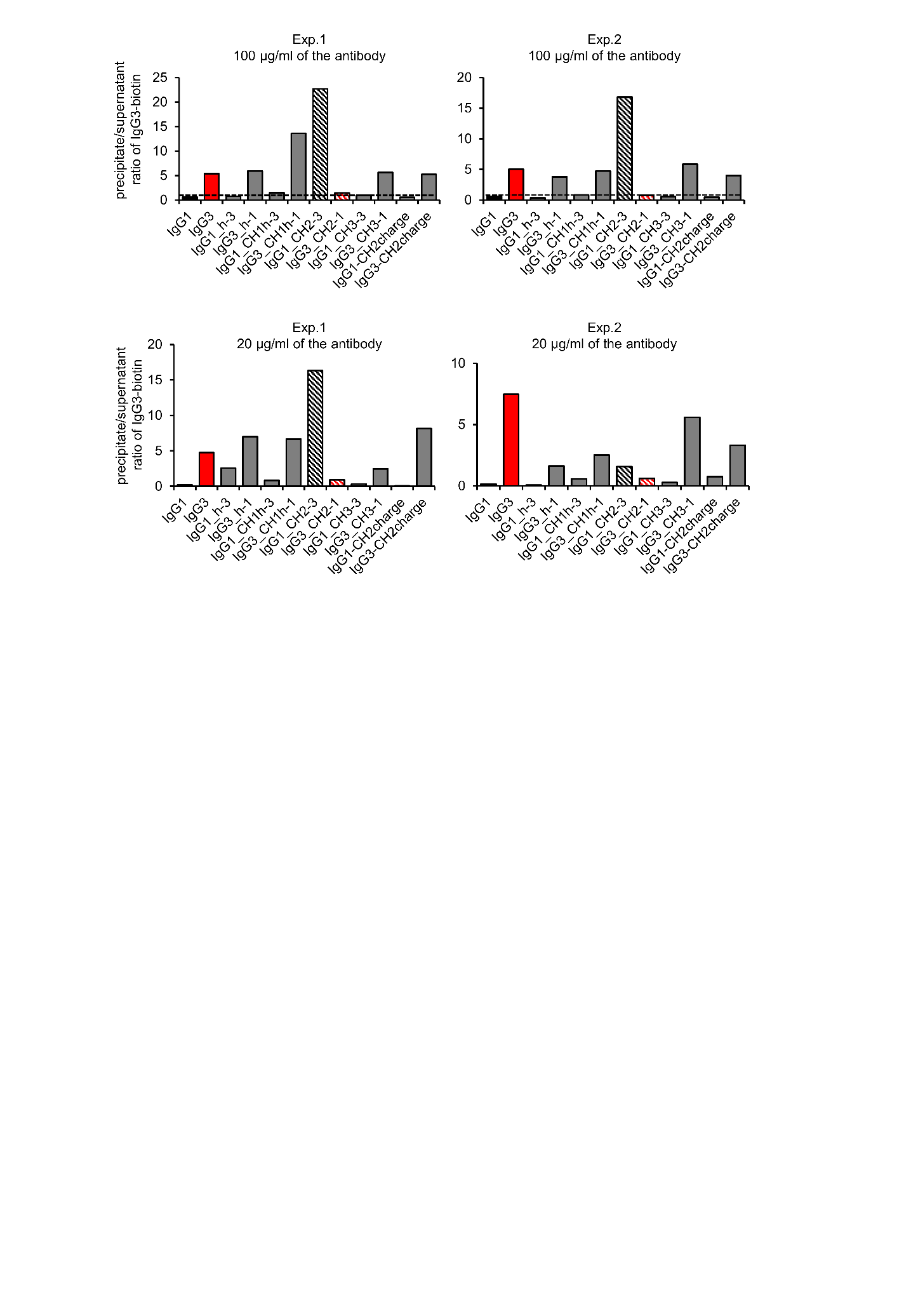
Supplementary Figure 3** – Oligomerization of the domain muteins. The charts present results from two independent experiments performed using 100 μg/ml and 20 μg/ml of the muteins.

*
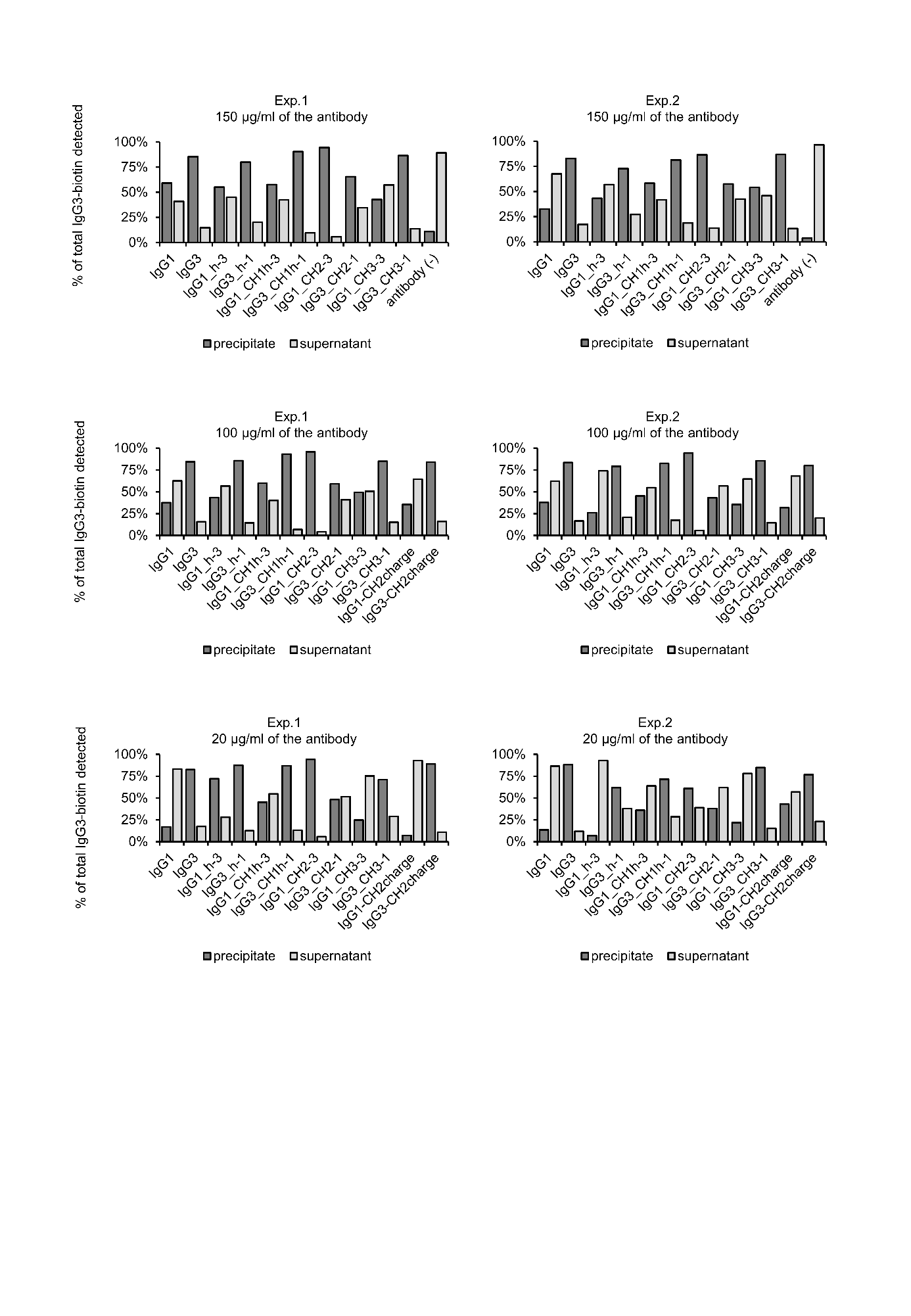
*

**Supplementary Figure 4** – Oligomerization of the domain muteins. The charts present a percentage of the total IgG3-biotin detected in precipitates and supernatants in two independent experiments.


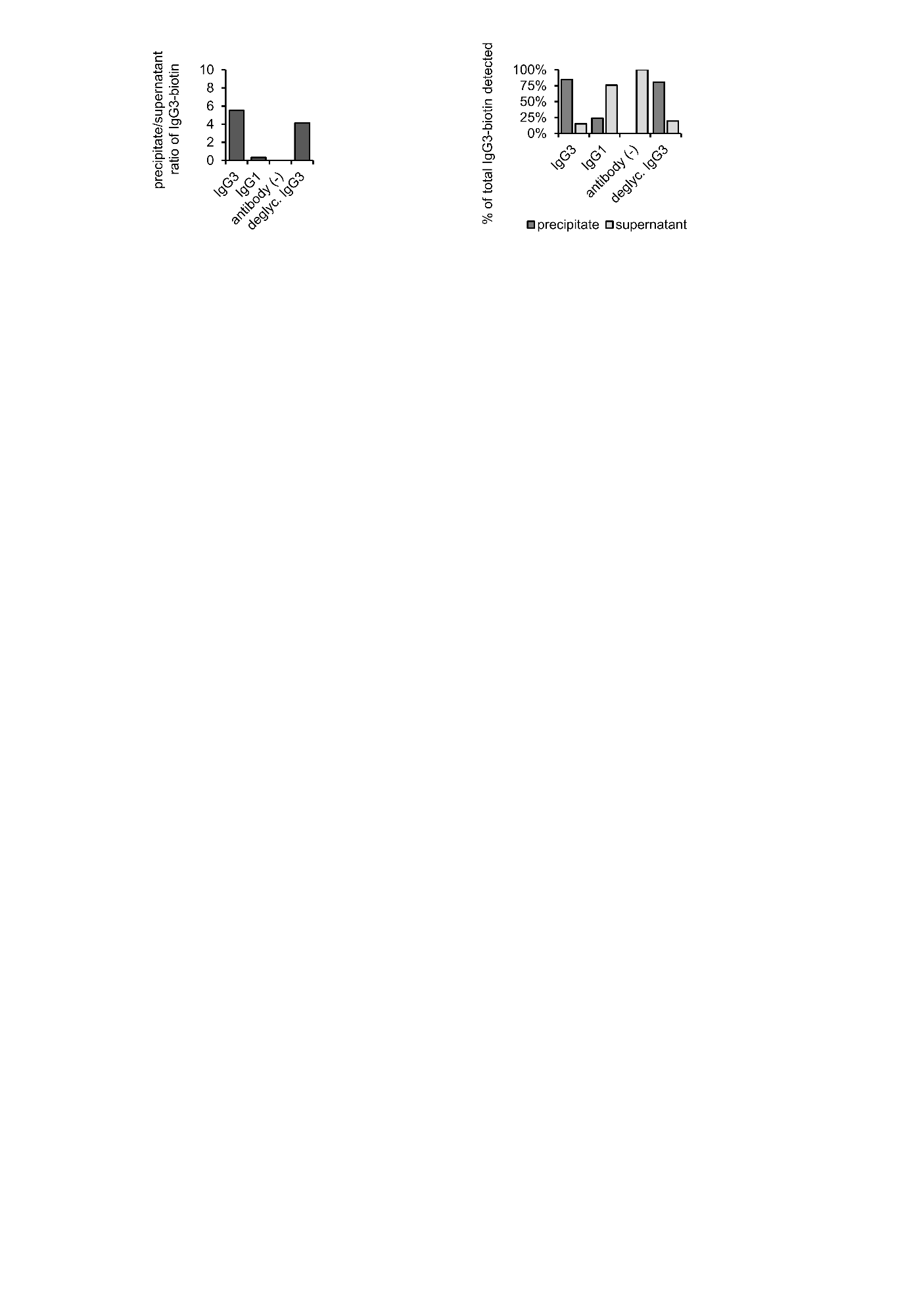


**Supplementary Figure 5** – Oligomerization of deglycosylated IgG3 (M18). The charts present results from an experiment performed using 100 μg/ml of the samples.

sp|P01857|IGHG1_HUMAN ASTKGPSVFPLAPSSKSTSGGTAALGCLVKDYFPEPVTVSWNSGALTSGVHTFPAVLQSSG

sp|P01868|IGHG1_MOUSE AKTTPPSVYPLAPGSAAQTNSMVTLGCLVKGYFPEPVTVTWNSGSLSSGVHTFPAVLQ-SD

sp|P03987-2|IGHG3_MOUSE -TTTAPSVYPLVPGCSDTSGSSVTLGCLVKGYFPEPVTVKWNYGALSSGVRTVSSVLQ-SG

.*. ***:**.*.. :.. .:******.********.** *:*:***:*. :*** *.

CH1

sp|P01857|IGHG1_HUMAN LYSLSSVVTVPSSSLGTQTYICNVNHKPSNTKVDKKV*EPKSCDKTHT----*CPPC--PAPE

sp|P01868|IGHG1_MOUSE LYTLSSSVTVPSSPRPSETVTCNVAHPASSTKVDKKI*VPRDCG--------*CKPCICT---

sp|P03987-2|IGHG3_MOUSE FYSLSSLVTVPSSTWPSQTVICNVAHPASKTELIKRI*EPRIPKPSTPPGSS*C-----PPGN

:*:*** ****** ::* *** * *.*:: *:: *: *

upper hinge core___

CH2

sp|P01857|IGHG1_HUMAN LLGGPSVFLFPPKPKDTLMISRTPEVTCVVVDVSHEDPEVKFNWYVDGVEVHNAKTKPREE

sp|P01868|IGHG1_MOUSE VPEVSSVFIFPPKPKDVLTITLTPKVTCVVVDISKDDPEVQFSWFVDDVEVHTAQTQPREE

sp|P03987-2|IGHG3_MOUSE ILGGPSVFIFPPKPKDALMISLTPKVTCVVVDVSEDDPDVHVSWFVDNKEVHTAWTQPREA

: ***:*******.* *: **:*******:*.:**:*:..*:**. ***.* *:***

sp|P01857|IGHG1_HUMAN QYNSTYRVVSVLTVLHQDWLNGKEYKCKVSNKALPAPIEKTISKAKGQPREPQVYTLPPSR

sp|P01868|IGHG1_MOUSE QFNSTFRSVSELPIMHQDWLNGKEFKCRVNSAAFPAPIEKTISKTKGRPKAPQVYTIPPPK

sp|P03987-2|IGHG3_MOUSE QYNSTFRVVSALPIQHQDWMRGKEFKCKVNNKALPAPIERTISKPKGRAQTPQVYTIPPPR

*:***:* ** * : ****:.***:**:*.. *:*****:**** **: : *****:** :

CH3

sp|P01857|IGHG1_HUMAN DELTKNQVSLTCLVKGFYPSDIAVEWESNGQPENNYKTTPPVLDSDGSFFLYSKLTVDKSR

sp|P01868|IGHG1_MOUSE EQMAKDKVSLTCMITDFFPEDITVEWQWNGQPAENYKNTQPIMNTNGSYFVYSKLNVQKSN

sp|P03987-2|IGHG3_MOUSE EQMSKKKVSLTCLVTNFFSEAISVEWERNGELEQDYKNTPPILDSDGTYFLYSKLTVDTDS

::::*.:*****::..*: . *:***: **: ::**.* *:::::*::*:****.*:..

sp|P01857|IGHG1_HUMAN WQQGNVFSCSVMHEALHNHYTQKSLSLSPGK

sp|P01868|IGHG1_MOUSE WEAGNTFTCSVLHEGLHNHHTEKSLSHSPGK

sp|P03987-2|IGHG3_MOUSE WLQGEIFTCSVVHEALHNHHTQKNLSRSPGK

* *: *:***:**.****:*:*.** ****

**Supplementary Figure 6** – Multiple sequence alignment of human IgG1, mouse IgG1 and mouse IgG3 constant regions. Residues highlighted in black, green and red are crucial for complement cascade activation by human IgG1 (Duncan and Winter, 1988;Tao et al., 1993;Idusogie et al., 2000). The fragments comprising green residues were swapped in the IgG1_ILGGP and IgG3_VPEVS muteins. Residue 322 (red) was mutated in IgG1_Arg322Lys and IgG3_Lys322Arg muteins.


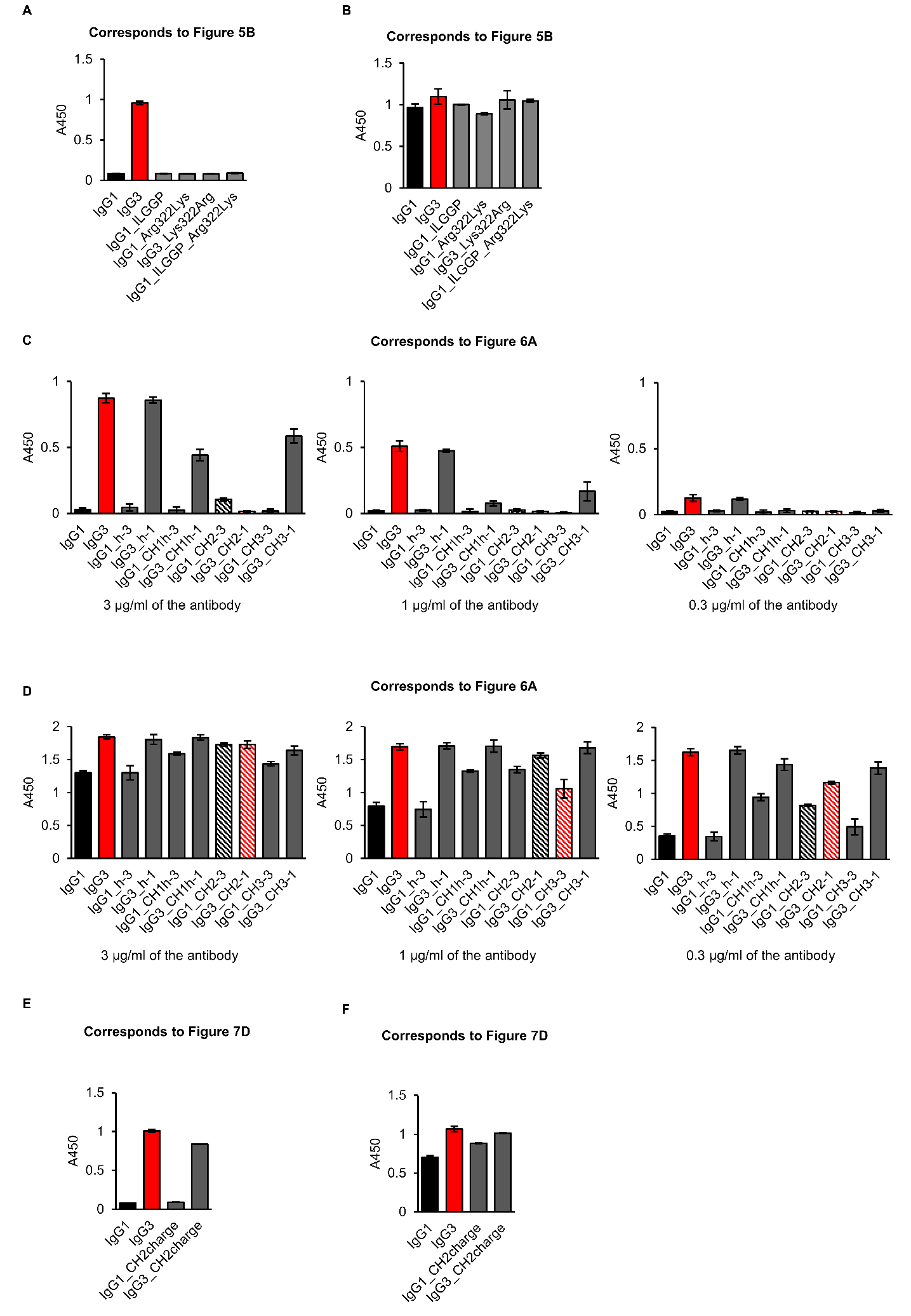


**Supplementary Figure 7** – Complement activation induced by the domain muteins. (**A**, **C**, **E**) C1q binding to the domain muteins analyzed using ELISA. Mean values and standard deviations from one experiment performed in duplicates or triplicates are presented. The data were used for calculation of normalized C1q binding showed in indicated figures in the main text. Representative results of two independent experiments are shown. (**B**, **D**, **F**) Binding of the muteins to plates coated with the B-antigen conjugated to BSA. The binding was analyzed using ELISA. The figure presents mean values and standard deviations from one experiment performed in duplicates. The data were used for calculation of normalized C1q binding showed in the indicated figures in the main text.


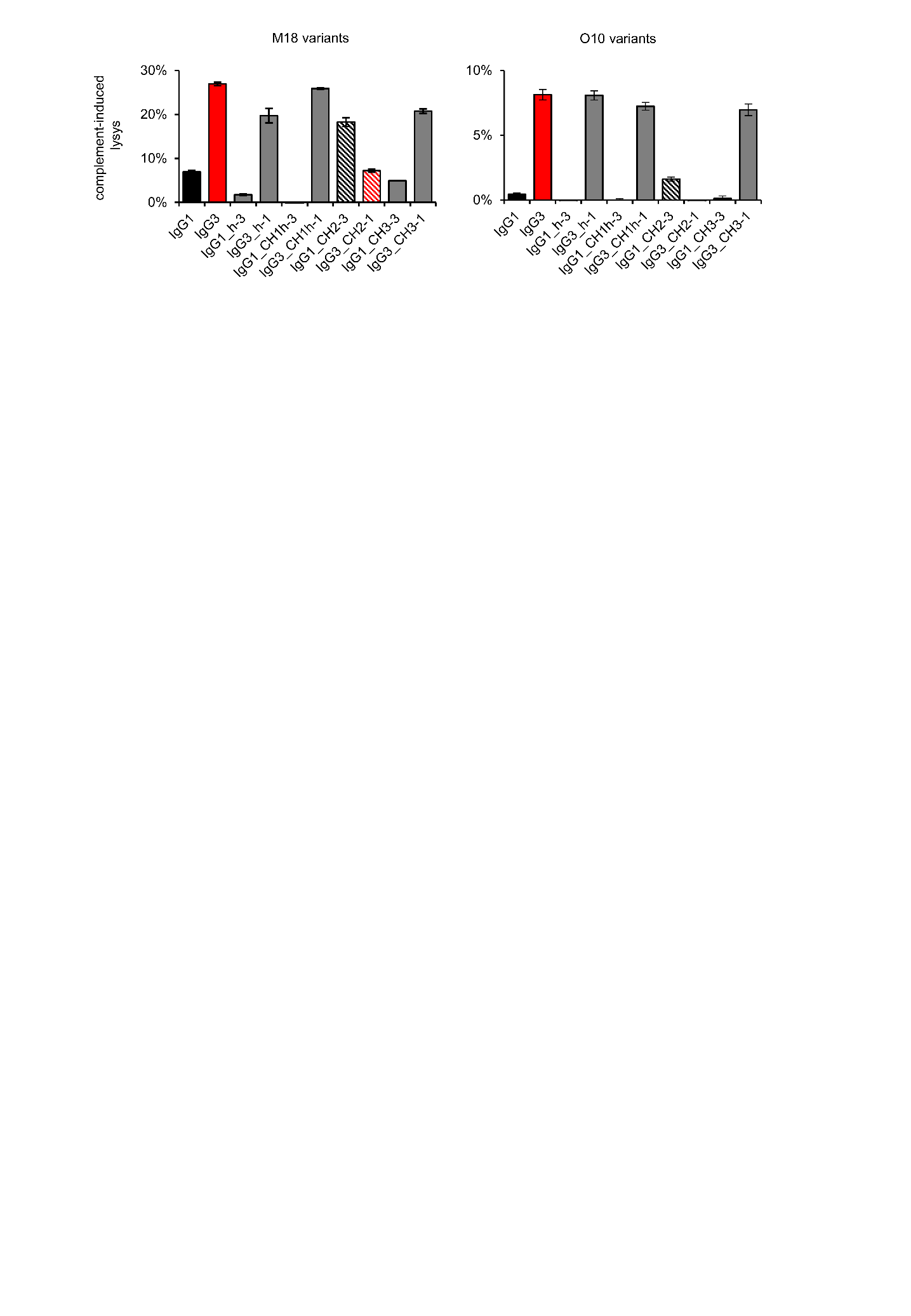


**Supplementary Figure 8** – Complement cascade activation by the domain muteins. Erythrocytes coated with 1.5 μg/ml of the muteins were incubated with complement serum. 100% lysis corresponds to water-induced lysis. The charts present mean values and standard deviation of duplicates from one experiment. Representative results of two independent experiments are shown.

.

IgG1 1 --VPEVSSVFIFPPKPKDVLTITLTPKVTCVVVDISKDDPEVQFSWFVDD 48

:....|||||||||||.|.|:|||||||||||:|:|||:|..|||||:

IgG3 1 GNILGGPSVFIFPPKPKDALMISLTPKVTCVVVDVSEDDPDVHVSWFVDN 50

IgG1 49 VEVHTAQTQPREEQFNSTFRSVSELPIMHQDWLNGKEFKCRVNSAAFPAP 98

.|||||.|||||.|:|||||.||.|||.||||:.||||||:||:.|.|||

IgG3 51 KEVHTAWTQPREAQYNSTFRVVSALPIQHQDWMRGKEFKCKVNNKALPAP 100

IgG1 99 IEKTISKTK- 107

||:||||.|

IgG3 101 IERTISKPKG 110

**Supplementary Figure 9** – Alignment of the CH2 domain sequences. Residues are color-coded as in Figure 6. Four residues swapped between IgG1 and IgG3 are highlighted in red.
